# Assessing the Risk of HIV and Hepatitis C among Internally Displaced Persons in Georgia

**DOI:** 10.5334/aogh.2671

**Published:** 2020-06-24

**Authors:** Joshua Elbaz

**Affiliations:** 1 Boston College, US

## Abstract

**Background:**

Georgia is leading one of the world’s first hepatitis C (HCV) elimination programs alongside interventions to combat a HIV epidemic concentrated among high-risk groups. Although progress has been substantial, neither strategy accounts for the nearly 150,000 internally displaced persons residing in collective centers (CC-IDPs) who are susceptible to deeply integrated risk environments that could promote infection. Achieving dedicated goals for HCV elimination and HIV suppression requires a clear understanding of the risks facing CC-IDPs.

**Objectives:**

This literature review aims to consolidate what is known about the socio-economic and physical/mental health status of IDPs living in collective centers in Georgia, and to assess their vulnerability to HIV and HCV in light of local and global epidemiological trends.

**Methods:**

Sources were compiled from journal publications, reports by government ministries and transnational organizations, and the Integrated Household Survey database (2009–2018; updated annually by the National Statistics Office of Georgia) through manual searches in PUBMED, Google Scholar and Search, ProQuest, and digital repositories of government offices.

**Findings:**

Reports indicate that CC-IDPs are more susceptible to poverty, poor living conditions, mental illness, disability, substance use, and in some cases infectious disease; although, the correlation is not always present and subject to variability. These factors were linked to increased transmission and acquisition of HIV/HCV in both displacement and non-displacement contexts abroad. The geographic concentration of HIV/HCV in areas with greater clusters of CC-IDPs, and shared characteristics with local high-risk groups, indicate the possibility of inordinate transmission among CC-IDPs in Georgia.

**Conclusions:**

The disproportionate prevalence of psychosocial and clinical harms among CC-IDPs testifies to the serious potential of a greater burden of HIV and hepatitis C. Going forward, targeted research is needed to inform interventions and clarify the health status of CC-IDPs in Georgia.

## Introduction

Forced displacement has been an enduring reality of Georgian statehood for nearly three decades. In the wake of major state conflicts between 1991–1993 (old caseload) and in 2008 (new caseload), the country hosts about 282,000 registered internally displaced persons (IDPs),^1^ constituting roughly 7% of Georgia’s total population [1]. While a narrow majority of IDPs have managed to live relatively seamlessly among the general population, the remainder continue to live in collective centers (CCs), which consist of various non-residential buildings – schools, unfinished buildings, hospitals, abandoned Soviet-era hotels, etc. – never designed for permanent housing, but have since been taken up as mass shelters for extended periods of time [2]. As of February 2019, 48% of the IDP population live in CCs, three-quarters of whom are old caseload IDPs [1].

Given their scale and unique risk environments, CCs constitute an important health context with several indicators pointing to the serious potential of greater burden of disease for the IDPs living in them. Sociological and public health reports on IDPs in collective centers (CC-IDPs) depict a population that is disproportionately vulnerable to a number of individual and environmental risk factors, such as sub-optimal living conditions, economic instability, trauma-related mental illness, substance use, and gender-based violence. These factors are not only deeply interconnected in complex feedback systems, but are also major determinants of HIV and hepatitis C (HCV) infection, and risky behavior that leads to the transmission of viral agents. Both diseases have been reported in concentrated epidemics among high-risk groups in Georgia: injection drug users (IDU), MSM, commercial sex workers, and incarcerated populations.

In response, Georgia has developed strategic plans that outline comprehensive, nationwide interventions to minimize and eliminate HIV and HCV, respectively. Georgia currently hosts one of the world’s first HCV elimination programs initiated in 2015, and is committed to achieving 90-90-90 target goals for HIV diagnosis, treatment, and viral load suppression. While tremendous progress has been made on both fronts, it is nevertheless difficult to situate the status of CC-IDPs because data on the prevalence and transmission of HIV and HCV on CC-IDPs as well as barriers, facilitators, and degree of participation in ongoing interventions is largely unavailable. It is thus worth reviewing the status of CC-IDPs and assess their vulnerability in order to inform future research and strategy both locally and globally.

## Methods

This review compiles peer-reviewed literature and reports by government ministries and international organizations spanning between 1999–2019, with a particular focus on post-conflict (2009) and post-election (2013) reporting. Because of limited reporting in consolidated databases and the widespread dispersion of documentation, manual searching was relied on to navigate through online resources, namely PUBMED, Google Scholar and Search, ProQuest, and digital repositories of government offices. Searches were framed by a reference to “IDP” or “internally displaced persons” in addition to “Tbilisi” or “Georgia”. The search was further elaborated with relevant modifiers, such as “mental illness”, “living condition”, “poverty”, “depression”, “substance use”, etc. References in materials were also considered for possible inclusion.

Independent analysis of data provided by the Integrated Household Survey (2009–2018), which has been updated annually by the National Statistics Office of Georgia since 2002 and includes a nationally representative sample of IDPs, was used to draw comparisons with the general population. The IHS, however, does not explicitly report on CC-IDPs, and relies on self-identification – whether respondents reply “yes” to being IDP – and not the official IDP registry of the government to confirm IDP status. Thus, the survey can miss well-integrated individuals who do not report themselves as displaced and are often on better socioeconomic standing [3]. The database is nevertheless comprehensive, up-to-date, and informative.

## Results

Titles and abstracts, when available, were screened for relevance in preliminary searches and followed by full text screening, after which 74 reports relevant to IDPs and the status of HIV and HCV in Georgia were included for analysis. Of them, four reports are periodically released which increases the total documentation (Table 1). The content was summarized in the following categories: living and economic conditions, physical and mental health status, substance use patterns, and current epidemiological trends of HIV and HCV in Georgia. Additionally, reviews concerning the vulnerability of IDPs to HIV and HCV globally were referenced to further inform the status of IDPs in Georgia.

### Living Conditions

Reports between 1999 and 2008 repeatedly uncover substandard living conditions of CCs with no clear indication of improvement over time. CCs during this period suffered from profound disrepair, unsanitary conditions, overcrowding, and lack of basic infrastructure.^1^ The prevalence of these issues, and their consistency across CCs, is sparsely indicated in two reported figures. The UN Office for the Coordination of Humanitarian Affairs (2003) estimated that 70% of CCs did not meet minimum living standards, and this is consistent with an internal review released by the Ministry of Internally Displaced Persons from the Occupied Territories, Accommodation, and Refugees of Georgia (MRA) in 2010 which concluded that out of roughly 1,600 CCs, 446 (27.8%) were too dilapidated to be repaired and needed to be closed for public safety, and out of an additional 1,140 (71.2%), 596 had the potential to be converted to durable accommodations and 544 were already converted [4]. This indicates that an overwhelming majority of CCs up to 2009 were not suitable for long-term housing or even posed an immediate threat to the livelihood of its inhabitants. Since then, improvements in living conditions for CC-IDPs have been tangible, but incremental, driven by a more effective state, clearer prioritization, and more dedicated resources. The living conditions of CC-IDPs today can be discerned by tracing this progress and assessing the difference.

The IDP State Action Plan passed in July 2008 and amended in 2009 outlined the transition of IDPs to durable, long-term housing solutions through the rehabilitation of CCs and idle buildings, the construction of new apartment blocks, financial assistance for mortgage and rent, and a privatization process transferring the ownership of rehabilitated, newly constructed, or government-purchased homes to IDPs [5]. The implementation of this scheme, however, has been inconsistent and error-prone, especially between 2009–2014 before rigorous standards for the accommodations process were implemented. This period was plagued by concerns regarding the quality of repairs and the practice of resettling IDPs. For instance, Transparency International Georgia (2011) and Amnesty International (2011) found that numerous refurbished buildings still did not meet the criteria for housing provided by international law [6,7]. Common problems cited in both studies included dampness and mold from leaky roofs and poorly installed windows, unfixed drainage and sewage infrastructure, unreliable supply of tap water and improperly installed tap water ports, damaged floors, unevenly fixed walls, and falling paint. In some cases, IDPs were provided with unfinished accommodations with incomplete bathroom ceilings and entrances. As a result, CC-IDPs express general discontent with their living conditions: a 2010 survey revealed that only 11% are completely satisfied, 20% somewhat satisfied, and 51% explicitly dissatisfied with the living space they inhabit [8]. Subsequent reviews conducted by the Public Defender’s Office (2010–12) note that “despite some progress, the findings of the monitoring process and individual complaints filed by IDPs show that the standard of living of IDPs has not improved” [4]. The MRA also acknowledges in their 2014 report that resources were mishandled and the lack of defined standards for durable housing allowed for negligence and oversight [9]. The lack of unified allocation practices, despite existing standards, also proved consequential for the resettlement process between 2009–2013. Issues included: offering families smaller households, splitting families, lack of infrastructure in resettlement areas, drastically extended work commutes, and forced evictions that cut IDPs from their social networks and sources of income, healthcare, and education [1,10].

The spatial distribution of CCs also raises concerns over isolation and social integration. Typically clustered closely together and concentrated on the outskirts of urban areas or remote locations, CCs impose a physical distance between CC-IDPs and the local population while promoting social interaction within CCs [11]. Because these areas are more geographically distant from cities and towns, a significant number of CC-IDPs are highly dependent on public transport, even though reliable public transport systems are difficult to find across the country. This compromises their mobility and creates varying degrees of isolation from local people, basic amenities, and employment and municipal services; the lack of community infrastructure in many settlements no doubt compounds these issues [12]. Thus, CC-IDPs often feel excluded from host communities and spend more of their time in the areas surrounding their residence with other IDPs [11,13,14]. These socialization patterns are reflected in strikingly homogenous social networks: Mitchnek (2009) found about 87% of their networks are composed of kin and other displaced, although no significant differences were seen in network size between CC-IDPs and the general population [15]. Social or spatial insulation and isolation can exacerbate marginalization, which in turn can affect employment, livelihood opportunities, mental health, and patterns of substance use.

At the request of international organizations, these concerns manifested in various amendments between 2012–2014 to the law on IDPs – which now grants full protection from forced evictions in premises under the legal ownership of IDP – and to the IDP Action Plan (first passed in 2008 and amended in 2009) to include minimum shelter and renovation standards, allocation guidelines, a purchase agreement, and standard operational procedures to guide the privatization process [5]. The introduction of these guidelines between 2012–14 marks a turning point in how progress on durable accommodations has been tracked. According to the MRA (2009–14), 33,349 IDP families have been considered settled, meaning that families were either given new or rehabilitated homes – some of questionable quality as mentioned previously – or financial assistance towards rent or privatization. Since then, the Public Defender’s Office (2015–2017) provides a more accountable and illustrative report on the progress of durable accommodation. An additional 5,272 IDP families were provided with durable accommodation – including 806 families relocated from collapsing buildings. While still prone to inconsistencies, these improvements appear to be more reliable. Taken together, over 52,000 IDP households still do not have durable accommodations, and of roughly 37,000 households considered settled, some reservation as to the durability of these solutions is warranted, especially among the 33,000 settled between 2009–2014 [4].

**Figure 1:**
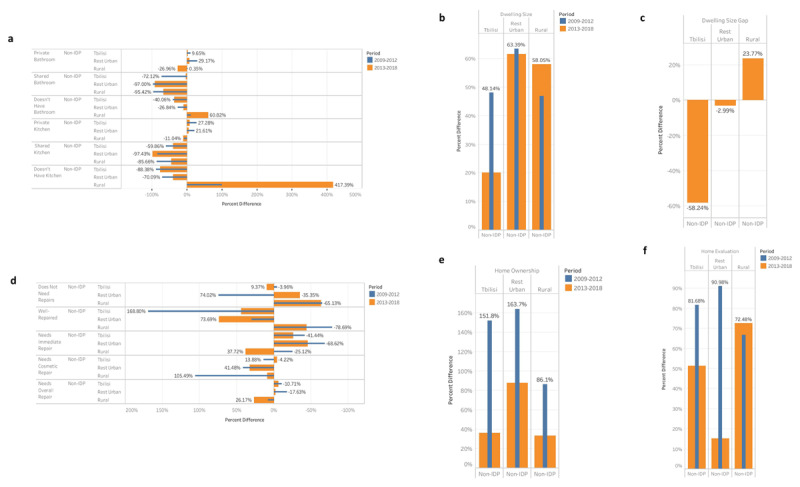
**Comparison of living conditions between IDPs and non-IDPs.** All measures are stratified by geography and two time periods representing the post-election period (2013–2018) and the post-conflict period preceding the election (2009–2012). (**a**) Percent difference of access to private or shared bathrooms and kitchens, or the lack of either in the respondent’s residence, between IDPs and non-IDPs. (**b**) Percent difference in dwelling size. (**c**) Change in the difference between IDP and non-IDP dwelling size over time, calculated as the percent difference of (**b**) between the two time periods. (**d**) Status of home repairs according to the urgency of repairs and the severity of repair. (**e**) Percent difference in home ownership. (**f**) Percent difference in the self-perceived value of the respondent’s home according to what they believed others would pay to purchase their home.

The IHS (2009–2018) also reveals positive trends in home ownership, dwelling size, and value (Figure 1). Across all geographic areas, IDP home ownership and value increased by 40–82% and 42–124%, respectively. The total dwelling size has remained relatively unchanged for IDPs in rural areas, with more substantial improvements in Tbilisi (25.81%) and other urban areas (8.82%). Nevertheless, by all measures non-IDPs fared significantly better than IDPs. Similar trends are also present with regards to access to bathrooms and kitchens – an important metric of hygienic living conditions – and the status of home repairs (Figure 1). A greater proportion of non-IDPs have a private bathroom and kitchen and do not share these facilities compared to IDPs. Despite serious inequalities in housing conditions between IDPs and non-IDPs, this gap appears to be closing over time. For instance, the difference in home valuations decreased by 30% and 76% in Tbilisi and other urban areas, respectively. Across all geographic areas, the difference in home ownership decreased by 53–115%, and in Tbilisi, the difference in dwelling size decreased by 28%.

### Economic Instability

CC-IDPs suffer from disproportionately high unemployment rates and low-income levels. According to the IHS (2009–18), non-IDPs earned more than IDPs in virtually every source of income by 1–910%, except for pensions, scholarships, and assistances where non-IDPs earned 41–60% less across the same time period. These gaps appear to be both opening (7–726%) and closing (4–98%) across various measures. Notably, the total income gap appears to be closing in Tbilisi and other urban areas, but growing in rural areas. This is consistent with several studies that identify lower income relative to the national average. Of these, Gachechiladze et al. (2013) is the only study that explicitly reports income levels for CC-IDPs in their cohort, over half of which were roughly equal to the national subsistence minimum (250 GEL) [16].

IDPs are the beneficiaries of several targeted assistance programs that can provide supplementary income.^1^ Over time, CC-IDPs especially have become increasingly dependent on social allowances which often constitute their largest source of income [10,17,18]. Interestingly, there are instances where social allowances remain the primary source of income despite IDPs being employed, suggesting that employed IDPs earn less [17,19]. This may be due to a number of deeply entrenched barriers to stable and self-sustaining jobs, namely the absence of previous work experience, limited access to land and financial institutions, failure to recognize the qualifications and demands of the labor market, lack of resources for vocational training and skill building, and the isolated and underdeveloped nature of many settlement areas which negatively constrains social networks.^1^ High levels of economic dependency also increase the risk of developing a psychological dependency, which can create a reinforcing loop that further entrenches CC-IDPs in economic instability. The prospect of benefit reductions can produce fear that compromises their desire to find employment. A lack of financial independence is especially impactful on the self-esteem of IDP men, who generally find it more difficult to secure employment, in part because of an unwillingness to do menial work or participate in vocational training that is not as pronounced in women. Aside from the cultural stigma associated with financial dependence, a poor understanding of the social allowance system and eligibility criteria might also contribute to misconceptions that prevents IDPs from taking jobs, particularly in the formal economy [2].

Unsurprisingly, numerous reports have revealed disproportionately high unemployment rates among IDPs, especially CC-IDPs, compared to the general population.^1^ Across all reports, IDPs exhibited unemployment rates 2–4 times greater than the general population. CC-IDPs appear to fare worse than those in private accommodations, and there does not appear to be a discernable difference between old and new caseload IDPs. According to data provided by the Georgian Statistical Office (2013), urban IDPs also appear to be 3.5 times more likely to be unemployed than their non-IDP counterparts [10]. This is consistent with the findings from the IHS demonstrating lower employment rates among IDPs compared to the general population: across all four employment measures – economically active, employed, hired, and self-employed – non-IDPs report 15–112% higher rates between 2009–16. This gap also appears to be increasing by up to 400%. Moreover, IDPs remain unemployed for longer periods of time compared to the general population; according to a 2013 World Bank study, the percentage of poor IDPs who have been unemployed for more than a year is double that of poor non-IDPs. Low-income IDPs are also three times as likely to have never worked before, indicating higher levels of discouragement and limited integration in social and professional networks. This again is consistent with IHS data. Between 2009–16, non-IDPs were unemployed less (20–61%), unemployed for shorter periods of time (10–82%), and more hopeful in their job search (48–75%). Whether these gaps are opening or closing over time is less consistent and appears to be split fairly evenly across all measures.^1^

Surveys by Tskitishvili (2005) and FAO (2009) are notable exceptions to findings on employment; both report similar employment rates between IDPs, the local population, and the national sample. The former found employment rates improving among IDPs from the early 1990s to 2005, even surpassing the general population (GP) with no major differences in informal and formal employment [19]. Similarly, the unpublished FAO baseline assessment found unemployment rates in some new caseload settlements was close to that of host communities [10]. Tskitishvili (2005), nevertheless, acknowledges that while IDPs appeared to be more employed, they earned significantly lower income from employment compared to the GP.

Low income and high unemployment are key drivers of poverty. Various indicators and indexes have been used to track poverty among CC-IDPs, namely: self-identification as poor based on income [6,16], ability to meet basic nutritional needs [6,13], ownership of productive assets or durable goods [6,20], home ownership [3,10,17], size and adequacy of living spaces [3,13], dependence on state benefits, and a multidimensional poverty index which considers demographics, education levels, employment status [3], and access to basic infrastructure [3,4,6,10,13,21].^1^ By several measures, poverty appears to be more concentrated among IDPs and more so among CC-IDPs, although some reports using IHS data suggest poverty rates between IDPs and non-IDPs are not significantly different [18].

### Health Risks

#### Mental Health

The psycho-social vulnerabilities of IDPs in Georgia have been well documented. Studies have largely focused on post-traumatic stress disorder, generalized anxiety disorder (GAD), depression, somatic stress disorder, and insomnia. Studies vary by sampling strategies, sample sizes, and investigative comparisons. Data on mental illness has been dissociated according to caseload (old vs. new), dwelling (CC vs. PA), gender, IDP status (IDP v non-IDP), and age (65+ elderly v other age groups). In some cases, data was not disaggregated and investigators simply looked at the prevalence of these conditions among the entire IDP study population (Table 2). Furthermore, studies have overwhelmingly relied on standardized patient health questionnaires which can indicate *symptoms* of mental health disorders, but are not clinical diagnoses.

**Table 2:** Mental health studies on IDPs in Georgia.

Year	Survey	Author	Survey Size	Sample Population	Data Stratification	Conditions Studied	Findings	Diagnostic Criteria

1995	Psychosocial Examination of Children and IDP Women – Victims of Military Conflict on the Territory of the Republic of Georgia’	OXFAM	650	Women and children		PTSD Migraine Sleep Disorders Depression	High rates of all studied conditions, including associated physiological symptoms (i.e heart and cardiovascular disease)	
2011	Insomnia in a Displaced Population is Related to War-Associated Remembered Stress	Tamar Basishvili Marine Eliozishvili Lia Maisuradze Nani Lortkipanidze Nargiz Nachkebia Tengiz Oniani Irma Gvilia Nato Darchia	105	Collective CenterFrom Abkhazia	Age Gender Socio-economic level Marital Status Employment Livelihood Satisfaction	Insomnia	Incidence of insomnia was high and strongly associated with war-related stress, frequency of nightmares, perceived stress, and depression. Good sleepers were also significantly younger	Physician- and certified specialist-conducted sleep interviews and medical examinations DSM-IV Beck Depression Inventory Insomnia Severity Index Perceived Stress Scale
2012	Aging in Displacement: Assessing Health Status of Displaced Older Adults in the Republic of Georgia	Namrita S. Singh, Nana Sumbadze, Paul Clayton Perrin, Judith Bass, George W Rebok, Courtland Robinson	899	Elderly (65+)	Gender IDP Status Age (>60) Settlement Type (Private Accomodation and Collective Center)	Depression General Anxiety Disorder	New caseload IDPs had higher depression and anxiety scores and lower health scores, but fewer exposures to traumatic experiences IDPs living in state-owned collective centers had higher levels of depression and more problems with slef-care, pain/discomfort, and being anxious or depressed, as well as a lower overall health score compared to IDPs living in private accomodations	Mini-Mental State Exam (MMSE), EuroQol (EQ-5D and EQ-VAS), Geriatric Depression Scale (GDS), Geriatric Anxiety Inventory (GAI), Harvard Trauma Questionnaire (HTQ), Armenian Dignity Scale (ADS), Alcohol Use Disorders Identifiction Test (AUDIT)
2014	Mental disorders and their association with disability among internally displaced persons and returnees in Georgia.	Nino Makhashvili, Ivdity Chikovani, Martin McKee, Jonathan Bisson, Vikram Patel, and Bayard Roberts	3025	Old Caseload (1193) New Caseload (996) Returnee (836)	Caseload Gender Age Trauma Exposure Cumulative Trauma Community Conditions Household Economic Status Physical Disability	Depression PTSD Anxiety	Prevalence for all conditions and comorbidity was significantly higher for old caseload IDPs relative to new caseload. Risk of all conditions increased according to gender (women are more vulnerable), older age, lower levels of education, greater exposure to traumatic events, poorer household economic status and community conditions, and increased levels of functional disability	Trauma Screening Questionnaire, Patient Health Questionnaire 9, Generalized Anxiety Disorder 7, WHO Disability Assessment Schedule 2.0
2014	Comparative Study of Psychological Well-Being and Posttraumatic Growth Indicators in IDP and Non-IDP Citizens of Georgia	Lili Khechuashvili	589	IDP and Non-IDP	IDP Status	Posttraumatic Growth Factor Well-Being	Measures were mostly the same between IDPs and non-IDPs, with the former scoring lower on opening new possibilities and the latter scoring lower on well-being scales with lower income	Posttraumatic growth inventory Psychological well-being scales
2015	Patterns of somatic distress among conflict-affected persons in the Republic of Georgia	Comellas RM, Makhashvili N, Chikovani I, Patel V, McKee M, Bisson J, Roberts B.	3600	Old Caseload (1200) New Caseload (1200) Returnee (1200)	Gender Trauma Exposure Cumulative Trauma Age Education Marital Status Household Economic Status	Depression PTSD Anxiety Somatic Distress	Somatic distress was significantly associated with older age, gender (women are over twice as likely), poorer household economic status, and increased levels of functional disability and exposure to traumatic events	Patient Health Questionnaire (PHQ-15), Trauma Screening Questionnaire, Patient Health Questionnaire 9, Generalized Anxiety Disorder 7, WHO Disability Assessment Schedule 2.0
2015	Health Service Utilization for Mental, Behavioural and Emotional Problems among Conflict-Affected Population in Georgia: A Cross-Sectional Study	Ivdity Chikovani, Nino Makhashvili, George Gotsadze, Vikram Patel, Martin McKee, Maia Uchaneishvili, Natia Rukhadze, and Bayard Roberts	3600	Old Caseload (1200) New Caseload (1200) Returnee (1200)	Health Service Utilization	DepressionPTSDAnxiety	Presence of mental illness were associated with higher rates of health service utilization	
2017	Coping strategies and mental health outcomes of conflict-affected persons in the Republic of Georgia	L. Saxon, N. Makhashvili, I. Chikovani, M. Seguin, M. McKee, V. Patel, J. Bisson, and B. Roberts	3600	Old Caseload (1200) New Caseload (1200) Returnee (1200)	Gender Coping Strategy	Depression PTSD Anxiety	Behavioral disengagement, substance abuse, and denial were significantly associated with symptoms of depression and anxiety. Substance abuse as a coping strategy was more significant among men The prevalence of mental health problems of all reported types was significantly higher in women than men	Trauma Screening Questionnaire, Patient Health Questionnaire 9, Generalized Anxiety Disorder 7, adapted Brief Coping Inventory

As for general patterns, women and elderly IDPs consistently exhibit significantly higher rates of all measured conditions. The rates of symptoms were nearly four times higher in older IDPs [22], and the risk of PTSD, depression, anxiety, and somatic distress increased by 1.5–5.5 times with age [20,23,24,25]. The difference is less dramatic between men and women, with the latter exhibiting 3–20% higher rates of symptoms. Interestingly, the trauma of displacement appears to affect men and women differently in surprising ways. Despite more pervasive mental illness among women, various reports suggest that women have been much more successful at adapting to everyday life [13,26,27].^1^ Excessive mental illness among the elderly, nevertheless, can relate to factors that are conflict and post-conflict related. Mental disorders may become entrenched over a sustained period of time with lack of access to adequate care and treatment [28].

Differences by dwelling type – CC or PA – appear to be indiscernible — 70.6% versus 71.2% (GAD) and 73.2% versus 68.3% (depression), neither of which were statistically significant. Differences by caseload are less clear. Makhashvili (2014) found higher rates of symptoms in old caseload IDPs, but JHU (2012) found the opposite trend [22,23]. The study population in JHU (2012) was also exclusively IDPs over the age of 65, as opposed to varied age groups in Makhasvili (2014). This might suggest that the difference between caseloads minimizes with older age and that the effect of displacement on the elderly is acute regardless of the length of displacement. In younger age groups, the difference between caseloads may be accounted for by greater hardship of displacement in the 1990s indicated in, for instance, higher exposure to traumatic events (i.e. witnessing murder) and poorer living conditions (smaller dwellings, less adequate housing, etc.) entrenched over longer periods of time [23,25].

In addition, ongoing impoverishment and poor living conditions may also exacerbate existing conditions. Insecurity of tenure, loss of home, job, and identity, the uncertainty associated with the collapse of the Soviet system (for older IDPs), lack of money, and a lack of optimism over the future impose enormous psychological strain on adults [13,29,30]. Interestingly, a study by FDHR (1997) found that over time, stress-related health problems and depression were becoming increasingly attributable to their post-displacement environment and less on conflict-induced traumatic experiences, citing growing emphasis on arduous living conditions, deep economic trouble, a perceived lack of interest from the Georgian government in their condition, and a growing sense of victimization, stigmatization, isolation, and segregation by local populations frustrated by their continued presence.

Khechuashvili (2014) and SC (2002) are the only studies comparing IDPs to the general population [20,31]. The latter, conducted in 2000, found that CC-IDPs had greater prevalence of depression symptoms compared to the general population (89.9% vs. 65.4%). Khechuashvili (2014) was not focused on the prevalence of mental health disorders, but rather the self-perception of psychological well-being. Surprisingly, there were no significant differences observed on the total score and subscales of psychological well-being, or four out of five factors on the post-traumatic growth inventory; IDPs scored lower on the New Possibility factor, which indicates lower confidence in the opening of new possibilities *in general*. Basishvili (2011) and Chikovani (2015) also report the prevalence of insomnia (41.4%), PTSD (23.5%), depression (14.4%), anxiety (10.9%), and comorbid symptoms (12.7%), but without comparisons between subgroups of IDPs [25,32]. In addition to age, gender, and status, a number of other demographic characteristics were significantly associated with symptoms of mental illness. Lower levels of education, poorer economic status and community conditions, greater cumulative trauma (experience of more traumatic events), particular traumatic events (witness of murder, physical abuse, among others), being widowed, and having a disability or long-term illness increased the risk of PTSD, depression, anxiety, and somatic distress symptoms by 1.5–10 times [23,24,25,33].

#### Physical Health

The literature available on the physical health of Georgian IDPs is alarmingly sparse, methodologically error-prone, and largely outdated as most sources date before 2006. While these studies may no longer be reliable or representative, they can still be informative.

Data provided by the Ministry of Health of the Abkhaz Government in Exile (MoHA) and the Center of Medical Statistics and Information (CMSI), while exhibiting major flaws in data collection and analysis systems, offers rare insight into differences in prevalence and incidence between IDPs and the general population. The morbidity patterns for IDPs between 1998–2002 were worse for all disease groups except for malignant neoplasms (cancer), congenital anomalies, and prenatal pathologies. The prevalence and incidence of infectious diseases were 3.6 and 2.9 times higher in adult IDPs than in the GP, respectively; prevalence was also higher for IDP children. It is unclear whether this data includes CC- and PA-IDPs or what regions and settings (urban or rural) the IDPs lived in [34].

Surveys conducted by SC (2000–02) and IFRC (1999) offer a slightly more reliable view of the health status of IDPs in the same time period. IRFC (1999) includes CC- and PA-IDPs as well as a local population in various regions throughout the country [20,34]. Similarly, SC (2000–02) compares CC-IDPs in western Georgia (Imereti and Samegrelo) to the local population. IFRC (1999) found acute and chronic illnesses were reported roughly equally between all three groups (CC-IDP, PA-IDP, and local population). Conversely, SC (2000–02) found that CC-IDPs had higher proportion of acute (41%) and chronic (33%) illness, as well as comorbidity (55%), compared to the GP (33%, 18%, and 36%, respectively). Furthermore, in concurrence with MoHA, SC (2002) also found higher rates of acute respiratory infections (28.7% vs. 23.3%), tuberculosis (0.4% vs. 0.1%), and injuries (0.9% vs. 0.7%), among others, compared to the local population. This is consistent with the National Institute of Tuberculosis and Lung Disease and CMSI (1998–2001) and Weinstock (2001): both report higher risk of TB infection among IDPs compared to the GP [34,35]. Notably, according to the Georgia Household Survey in 2001, household size and poverty status was a key indicator for the likelihood of reporting acute illness or an episode of chronic illness: poor households with more members are less likely to report acute illness. Because IDPs tend to be poorer and live in larger households, the gap in incidence and burden of illnesses between IDPs and the general population may be even higher than reported by the IFRC (1999) and SCF (2002) [34]. Available data also indicates that physical disability is more common among IDPs than the GP, but reporting has been inconsistent.^1^ Furthermore, unsanitary and deteriorated living conditions, poverty, mental illness, and lack of opportunities constitute additional risk factors associated with vulnerability to cardiovascular and infectious disease – primarily blood- and water-borne infections. Notable outbreaks of intestinal, hepatitis A, and typhus infections in CCs have been reported in Khoni Military Dwelling, Kutaisi Sanatorium “Khvamli”, and Zugdidi between 1996–2001 [34]. Studies also report elevated risk of STIs and pelvic inflammatory disease among IDP women, as well as a broader vulnerability to HIV: while constituting about 5.5% of the total population in 2006, IDPs accounted for 8.9% of PLHIV in Georgia [13].

It is important to note the degree of healthcare coverage available to CC-IDPs and their utilization of services. Despite concerning health outcomes, IDPs are entitled to expansive healthcare benefits within Georgia’s current universal healthcare coverage structure. Compared to the general population, CC-IDPs are more aware of the universal coverage system but are also largely uninformed on what services are covered, programs they can participate in, and whether they should expect to pay and in what amount for various treatments and tests. Barriers to healthcare utilization are multi-dimensional and help account for disparities in outcomes despite greater coverage [25,36,37].

#### History of Substance Use

Reports indicate high levels of alcohol consumption and tobacco use. A study identified 71% of CC-IDP men and 16% of women were current drinkers, of which 28% of men and 1% of women were classified as having at least hazardous alcohol use, in addition to 12% of men and 2% of women as episodic heavy drinkers [38]. Both measures were significantly associated with those who experienced injury, and the likelihood of hazardous drinking increased with greater cumulative exposure to trauma and symptoms of depression. A separate study identified 47.4% of CC-IDPs smoked and 70.9% of current smokers were heavy smokers. Nicotine dependence was also high and significantly associated with PTSD, depression, and older age. Conversely, hazardous and episodic heavy drinking were more closely associated with younger age groups (30–49) [39]. In both studies, old caseload IDPs demonstrated stronger patterns of abuse.

It is also critical to note both the high prevalence of nicotine and alcohol dependence *and* its strong correlation to mental health disorders and social conditions. Substance use appears to be a significant coping strategy among CC-IDPs with symptoms of mental illness, although the substances used and frequency of consumption is unclear [33]. Injection drug use (IDU) among Georgian IDPs is unknown. There are, however, several aspects to consider. First, what has been recorded over time is a number of testimonies of the IDP experience and life in CCs that recount significant drug use among middle-aged men, especially old caseload IDPs [34]. This is consistent with reports from 1998 and 2004, which documented a major increase in drug use throughout the country in the late 1990s, which is also coherent with the statewide instability during that period [40,41]. Second, quantitative reports on the prevalence of IDUs throughout Georgia have been conducted with some frequency, with some including an option to indicate IDP status in their survey questionnaire. While this has produced some data on IDP drug use, the sample sizes are far too small and geographically disparate to be representative [42,43,44]. It is important to also consider the social and cultural barriers to accurately surveying the IDU population. Heavy stigmatization against drug injection makes participants highly reluctant to share their experience out of fear that their family or community will learn of their status, a problem that is often compounded in CCs because of close living proximity and tighter social circles that can compromise anonymity [41,44]. These general population surveys, nevertheless, do capture a significantly growing IDU population (Table 3). Global AIDS monitoring data published by UNAIDS estimates the size of PWID population in Georgia to be 53,000 in 2017, a significant and steady increase since monitoring began. Prior studies from 2009, 2012, and 2014 roughly estimate the number of IDUs to be 40,000, 45,000, and 49,700, respectively.

**Table 3:** Population size of injection drug users in Georgia.

Survey	Year	Survey Size	Number of Cities Reviewed	Estimated Number of Users	Upper limit	Lower Limit	Method Used

The Drug Situation in Georgia	2003	6,107		50,000			Estimate based on Georgian Research Institute on Addiction (GRIA) database
The Drug Situation in Georgia	2004	14,400		80,000			Estimate based on Georgian Research Institute on Addiction (GRIA) database
Estimating the Prevalence of Injection Drug Users in Five Cities in Georgia	2009	1,127	5	40,000	41,062	39,000	Multiplier Method
Estimating the Prevalence of Injection Drug Users in Georgia	2012	1,791	6	45,000	45,524	44,434	Multiplier Method
Population Size Estimation of People Who Inject Drugs in Georgia	2014	1,951	7	49,700	50,192	49,208	Multiplier Method and Network Size Estimation Method
Population Size Estimation of People Who Inject Drugs in Georgia	2016	1,515	7	52,500	56,000	52,000	Multiplier Method and Network Size Estimation Method

Reports demonstrate that IDUs generally have a low socio-economic status: the vast majority of participants reported being unemployed, which varies from 51.3% in Gori to 73.2% in Kutaisi, and 51% of IDUs mentioned having a monthly income of less than 300 GEL. Of the entire sample (2037 participants), 247 IDUs reported having a permanent job, with the lowest proportion of 5.6% and the highest of 18.7% in Zugdidi and Telavi, respectively. The highest proportion of students was found in Tbilisi (1.2%). Every third participant mentioned having an average monthly income of 100–300 GEL across all seven survey sites. Every fifth respondent had an income of less than 100 GEL and the same proportion has a monthly income higher than 500 GEL (21%) in the combined sample [44].

There is no data on the mental health of IDUs in Georgia, so a parallel cannot be drawn reliably in that respect, although studies suggest that mental illness could pose a major barrier to treatment entry and effectiveness [45,46]. Emerging literature also describes a similar association between forced-migrant populations and substance use [47,48,49]. In effect, populations displaced by conflict have exhibited a range of substance use problems with various substances: alcohol [48,50], opioids [51,52,53], and benzodiazepine [54]. While evidence of continuation or inflation of pre-displacement patterns of use was present in some settings [48,53], others report new onset of use post-displacement [55]. Studies are also constrained by under-reporting due to stigmatization and a lack of methodological details and comparative populations [56]. Findings on patterns of substance use among displaced populations are, nonetheless, heterogenous, which may reflect regional differences that are influenced by a combination of local factors [56,57] (macro-economic changes [58], limited alternative livelihoods [53], poor governance [59], and setting (in transit or in resettlement, urban or rural) [55,60]) and individual risk factors (gender [48], exposure to war trauma [61,62], impoverishment, marginalization and discrimination [53,60], and co-existing mental health problems [45,46]).

#### Displacement and Infection Globally

A growing body of literature recognizes displacement contexts as important risk environments for the development of HIV/HCV [51,63,64]. As the Declaration of Commitment on HIV/AIDS (2001) states, “populations destabilized by armed conflict… including refugees, internally displaced persons and in particular, women and children, are at increased risk of exposure to HIV infection [65].” Several publications have indicated a direct association between conflict and increased HIV transmission, although to varying degrees [66,67,68,69]. In Pakistan, IDPs were reportedly overrepresented with respect to HCV prevalence [70,71]. Separate lines of evidence support the association between displacement and vulnerability to infection. Social networks are often destroyed or disrupted and those institutions that normally protect and support people are no longer in place or are ineffective [72]. Restricted geographic and social mobility can lead to social insulation, which may promote social relationships with other high-risk individuals if prevalence is significant throughout the community. Those in shared accommodations might also share personal equipment (i.e., razors, toothbrushes), which can increase the risk of HCV transmission [73]. Access to harm reduction resources – condoms or sterile equipment – may be scarce or compromised by other priorities in displacement settings [74].

**Figure 2:**
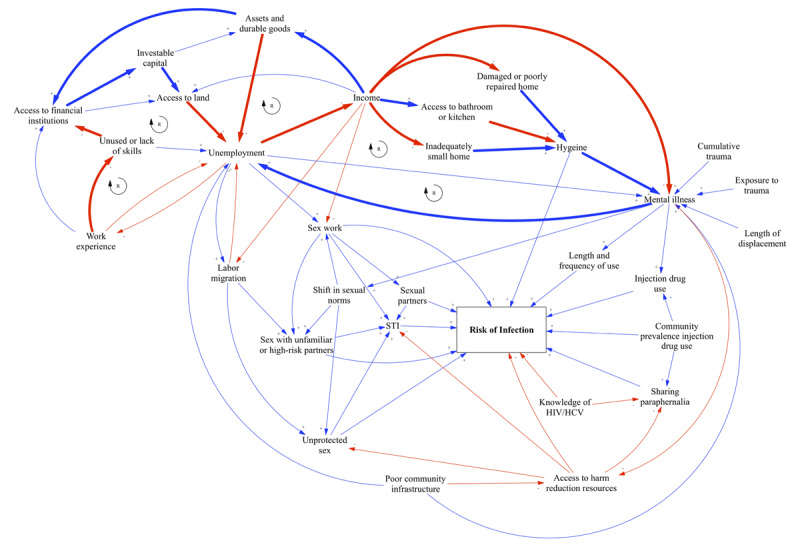
**Causal loop diagram of factors that contribute to the risk of HIV/HCV infection.** The diagram represents an informative, but not exhaustive, illustration of how various individual and environmental factors influence one another and the risk of infection. Blue and red arrows represent direct and indirect relationships, respectively. Reinforcing loops are signified, and further marked by bolded, thick arrows.

The breakdown of traditional sexual norms in conflict situations can also increase HIV exposure, but it is also invariably entangled in a number of psychosocial vulnerabilities such as substance use and mental health [74]. For instance, IV drug use appears to increase risky sexual behavior in several ways: having more sexual partners per year [75], transactional sex for drugs [76], having sex with unfamiliar or high-risk partners, and not using condoms [77,78]. In some studies, IDUs also appeared to perceive a lower risk of transmission through sexual contact than through unsafe injection practices, which led to risky sexual behavior [79,80]. Trauma and conditions that give rise to mental illness can contribute to socially isolated and financially unstable lifestyles [81,82], which can increase risk through the exchange of sex for money, shelter, drugs, and other goods [83]. In other words, socioeconomic vulnerabilities – poverty, poor living conditions, family conflict, mental illness, etc. – are often entangled with risky behavior in complex reinforcing patterns (Figure 2).^1^ Taken together, mass population movement and resettlement in temporary locations have been tied to poverty and unemployment, labor migration, sexual violence and abuse, increased drug use, lack of health infrastructure and education or awareness, overcrowding in settlement areas, poor health facilities, malnutrition, unhygienic food and water, poor sanitation, and mental illness, which are all major risk factors to blood-borne and sexually transmitted diseases [21].

Nevertheless, it is critical to acknowledge that IDP status does not necessarily translate to vulnerability, and vulnerability does not necessarily translate to infection or disproportionate burden of disease. Data collected during and after conflict is subject to bias and should be weighed appropriately [72,84]. Whether or not displacement affects HIV/HCV transmission depends on a number of context-specific factors. For instance, destroyed infrastructure that prohibits travel and reduces access to the host population, while certainly contributing to marginalization, can also be protective if it limits access to high-prevalence urban areas or hinders communication between high-risk groups. The duration of conflict and the length of displacement can also affect interaction between the two communities and contribute to further isolation. Thus, the prevalence of HIV/HCV in the local population prior to displacement and the level of interaction between the two populations are key environmental factors. Furthermore, collective centers are also more readily targeted by programs and local organizations aiming to improve access to education, health, and social services. Some studies have noted better preventive and curative health services in post-emergency refugee camps than the surrounding local community, even reporting lower HIV rates among displaced communities relative to the host population [72].

#### Georgian Context

How do the vulnerabilities of IDPs align with the current public health context in Georgia? Incidence of HIV in Georgia has grown since 2000 – 16.9 new cases per 100,000 people in 2017, compared to 6.7 (2010) and 2.0 (2000) [85] – but has stagnated and moderately decreased since 2012. The recent reduction in new cases reflects a combination of preventative strategies, which have increased health education and access to harm reduction resources, and targeted clinical interventions that have increased reporting and treatment enrollment over time. Progress toward the UN 90-90-90 target for HIV is also notable. Between 2011–2015, the proportion of diagnosed persons increased from 46% to 61%, antiretroviral (ART) coverage among diagnosed persons increased from 46% to 62%, and the proportion of virally suppressed patients among those on ART increased from 74% to 85% [86]. However, late diagnosis is a persistent problem through to 2017 where about 48% of the estimated number of infections were reported [87]. The effectiveness of these strategies, nevertheless, largely depends on their ability to incorporate high risk groups where HIV is concentrated. More specifically, while the national prevalence in adults aged 15–49 is about 0.4%, the rate is significantly higher in commercial sex workers (0.9%), MSM (16.2%), and IDUs (2.3%) [88]. In 2010, 46.7% of cases where transmitted through IDU, 43.3% by heterosexual sex, and 4.8% by homosexual sex. By 2016, these figures have drastically changed: 30.3% IDU, 51.5% heterosexual, 16.8% homosexual [87]. The epidemiology of HIV is transitioning from primarily drug-based to sex-based as interventions have effectively addressed the former.

In 2015, HCV prevalence was significantly higher in the general population (5.4%) compared to HIV, although similar epidemic levels were also present in high risk groups: anti-HCV prevalence rates were 66.5% in IDUs, 42.0% in those previously incarcerated, and 11.9% in those with more than two lifetime sexual partners. Other significant risk factors included urban living, unemployment, having tattoos, receiving more than one medical injection in the past 6 months, and having received blood transfusions [89]. Geographically, the highest rates of HCV were reported in Samegrelo-Zemo Svaneti (10.9%), with elevated rates also recorded in Shida Kartli (7.3%), Tbilisi (9.4%), and Imereti (7.5%), all regions that also host the greatest number and concentration of CCs [89]. These figures have significantly declined since the start of the HCV elimination program. Roughly two years after the start of the program in 2017, about 29.3% of the estimated 150,000 individuals living with HCV were diagnosed and of those, 77% were treated and 95% cured [90]. By 2019, over 54,000 have initiated treatment – 93% of whom have completed their treatment – and more than 1.5 million Georgians have been screened [91]. Nevertheless, it appears some financial, social, and educational barriers are constraining enrollment and continued treatment.

## Discussion

This study draws together evidence on the extent to which CC-IDPs in Georgia are uniquely vulnerable to HIV and HCV infection by examining disparities in living conditions, economic status, patterns of substance use, and physical and mental health, while also referencing the global context of displacement and infection. While these factors do represent determinants of health, a truly representative measure of risk requires quantitative and qualitative data on HIV and HCV acquisition. Without this information, our analysis is limited to inferences on the distribution of risk. Secondly, data available on CC-IDPs in Georgia is often methodologically inconsistent and error-prone, making it difficult to assess their reliability and comparability. This difficulty is compounded by the fact that CC-IDPs are an incredibly heterogenous group whose experience within CCs can differ drastically, thus resisting broad generalizations.

Nevertheless, available data on the livelihood of CC-IDPs suggests that they are at *relatively* greater risk compared to the general population. The risk, however, may not be uniform but concentrated in subgroups of CC-IDPs according to local and individual risk factors. Geographically, the risk is likely significantly higher in regions with greater prevalence, which can be divided along east and west Georgia with the latter generally exhibiting higher prevalence. Samegrelo-Zemo Svaneti in particular, a region in west Georgia bordering Abkhazia, has a high prevalence of HCV and is also home to the largest number of CC-IDPs outside of the capital region of Tbilisi. The risk of HIV is not as adherent to this pattern since the prevalence of HIV is very low in the general population.

Old caseload CC-IDPs may also be at greater risk owing to their overrepresentation in CCs. As mentioned earlier, the conditions of CCs underwent a shift post-2008 that prioritized both new housing for new caseload IDPs and an overhaul of existing CCs housing old caseload IDPs, a supermajority of which were not suitable for long-term housing or posed an immediate risk to their inhabitants. This transition is still largely incomplete and has been plagued by a number of shortcomings to the detriment of IDPs. Thus, old caseload IDPs are still more present in CCs where living conditions are more prone to overcrowding, disrepair, unsanitary conditions, or lack of infrastructure. This can increase the risk of exposure to contaminated material, as was the case with outbreaks of hepatitis A and typhus. Protracted displacement in substandard living conditions can also contribute to other social determinants of health that may influence risky behavior, such as greater rates of mental illness in old caseload IDPs. Overcoming housing insufficiencies can also drain financial resources away from productive investments and perpetuate poverty, which can encourage risky behavior.

A national seroprevalence study conducted in 2015 found unemployment to be a significant risk factor to HCV infection, and unemployment among IDPs – especially CC-IDPs – is consistently worse than the general population [89]. Thus, the dramatic economic disparity between CC-IDPs and the general population may be a real source of greater risk. Importantly, it is not clear how the size of CCs affects economic conditions and other livelihood domains. CCs can range from a handful of families to over 100 families, which can have a drastic effect on infrastructure and socialization that subsequently affects other aspects of livelihood.

The same seroprevalence study also reported that anti-HCV prevalence was three times greater in men than in women [89]. This disparity is likely present among IDPs; however, the greater burden of mental illness among IDP women may disrupt its severity. Mental illness has been linked to increased risk of infection, validating the possibility that IDP women may be at greater risk than non-IDP women [92]. A similar risk for HCV was recorded among older age groups, which may also be compounded in older IDPs by greater rates of mental illness. Nevertheless, mental illness has not been studied as an HCV-associated risk factor in Georgia, so it unclear to what extent IDP women are affected.

Information on the most direct contributors to infection in Georgia are unavailable for CC-IDPs or IDPs in general. For instance, injection drug use is a primary determinant of infection for both HIV and HCV, but its prevalence is unclear among IDPs. There is some evidence to suggest that CC-IDPs may be at greater risk of engaging in injection drug use. For one, several studies have found other forms of substance abuse (nicotine and alcohol) strongly associated with coping mechanisms for trauma or mental illness and discontent, which are disproportionately present among CC-IDPs. Second, there are characteristic overlaps between CC-IDPs and IDUs in the general population, namely economic instability. Third, there are a number of historical testimonies of injection drug use among older men in CCs, although primarily before 2004; they are, nevertheless, the most direct evidence of IDU among CC-IDPs and validate the possibility. Lastly, there is global precedent for displacement contexts contributing to IDU. Medical injection is also a risk factor for HCV transmission in Georgia, and CC-IDPs tend to report higher rates of chronic illness that could contribute to greater hospitalization and medical intervention.

HIV in Georgia has transitioned over the past several years from a primarily drug-induced epidemic to a sexual one, with hetero- and homosexual intercourse accounting for nearly two-thirds of new cases [85]. The former is still likely linked to unsafe sex between partners already infected through IDU rather than individuals from the general population. It is unclear the extent to which sexual transmission of HIV is taking place in CCs, especially without data on IDU. The degree of education and awareness of harm reduction practices is also unclear. HCV is rarely transmitted sexually and does not adhere to this pattern of activity.

The extent to which life in CCs are protective against HCV and HIV infection depends on local transmission patterns [72]. CCs are often concentrated together and pose a number of barriers to communication and interaction with host populations outside of the IDP community. Consequently, often times the social networks of CC-IDPs are constrained and homogenous, consisting mostly of immediate family members and other CC-IDPs. Thus, if the host population contained high-risk groups or had a high prevalence, then the insulating nature of CCs could protect against interactions that could lead to infection or the formation of risky behaviors. This may be most relevant to CCs around major urban centers – i.e. Tbilisi, Kutaisi, Batumi, etc. – where prevalence is generally higher and high-risk groups are more present. However, in CCs where prevalence is already high and risky behavior is conducted, their insulating effect could be a significant liability by fostering interaction that leads to infection.

Compromises in these livelihood domains – living conditions, economic status, physical and mental health, and substance use – pose serious direct and indirect threats to transmission, and this is consistent in displacement contexts globally. Nevertheless, the ongoing HCV elimination program has almost certainly played a significant role in managing infection since it formally began in 2015. The six-point strategic plan has employed initiatives to: (1) promote advocacy, awareness, and education, (2) prevent HCV transmission, (3) identify persons infected with HCV, (4) improve HCV laboratory diagnostics, (5) provide HCV care and treatment, and (6) improve HCV surveillance [93]. These efforts have been deployed both at a national scale and among targeted groups for micro-elimination, leading to substantial progress in diagnosing, treating, and curing HCV. Although available material on IDPs does not account for the impact of the elimination program, it has inevitably engaged IDPs; however, to what extent relative to other groups is crucially undetermined.

Globally, this challenge is familiar. The number of IDPs from state conflicts is increasing across the world – many of whom exhibit the same disparities seen in Georgia – alongside a global HIV and HCV threat. Accepting the challenge of United Nations Sustainable Development Goal 3, which aims to “ensure healthy lives and promote well-being for all at all ages”, requires an approach that recognizes the impact of IDP status on health and works to integrate this group with ongoing and future disease interventions.

## Conclusion

Poor living conditions, economic instability, mental and physical illness, and substance use are conditions that not only overlap and exacerbate one another, but also constitute determinants of HIV and HCV infection that may prove consequential on the burden of disease. The widespread presence of these individual and environmental risk factors among CC-IDPs in Georgia raises serious concerns as to whether they could be disproportionately burdened by HIV and HCV. Despite the fact that CC-IDPs have been identified as particularly vulnerable in the past, there is no reliable or updated public health data on HIV/HCV in Georgian IDPs, let alone CC-IDPs. Moreover, broader patterns of HIV/HCV prevalence and risky behaviors in Georgia appear conducive to promoting transmission in acutely vulnerable IDPs, although improvements in diagnosis, treatment, and other protective factors may also positively counterbalance those effects.

To achieve the goals outlined in the national HCV and HIV strategic plans, reliable documentation on this group is critically needed. Moving forward, IDPs should be considered as a key demographic in future HCV-related field research and should be the focus of new field research to generate representative data on the burden of disease and associated risk factors in order to inform targeted interventions. IDP status should also be integrated as a variable in existing health surveillance systems – the national HCV screening database, population-based cancer registry, electronic registers for maternal and child health, and HIV/TB databases – which can immediately and cost-effectively produce robust health data on IDPs who have already entered Georgia’s healthcare system. As the HCV elimination program shifts from a population-wide approach to smaller-scale groups to diagnose the remaining pockets of infection that have not yet been identified, CC-IDPs should be considered as a potential candidate for micro-elimination. Given that CC-IDPs constitute nearly 3% of the general population and live in well-defined, high-density areas, a targeted effort to screen each settlement and link positive cases to care could be a cost-effective approach to uncovering hidden cases, especially if informed by existing surveillance systems which can readily prioritize settlements according to their size and screening rate.

## Additional File

The additional file for this article can be found as follows:

10.5334/aogh.2671.s1 Appendix. A more detailed examination of referenced material for each section. Appendix also includes additional analysis of IHS data.

## Footnotes

See appendix.
