# Supplementary material for: Assessing the Risk of HIV and Hepatitis C among Internally Displaced Persons in Georgia
Source: Ann Glob Health. 2020 Jun 24;86(1):66. doi: 10.5334/aogh.2671 (PMC7319078; doi:10.5334/aogh.2671)
Supplement: Appendix. — A more detailed examination of referenced material for each section. Appendix also includes additional analysis of IHS data. [file agh-86-1-2671-s1.pdf]

Appendix

Table 1: Summary of Literature on IDPs in Georgia

Year	Title	Author	Type of Publication	Publisher	Methodology	Survey Sample Size	Sampling Strategy	Relevant Themes	Topics Covered
1999	Rapid Assessment of Injection Drug Use and HIV in the Republic of Georgia	Wouter De Jong, Tea Tasgarelli, Erik Schouten	Journal Publication	Journal of Drug Issues	Meetings with authorities, professionals, and other key informants<br><br>Interviews former and current injecting drug users (including three with HIV)<br><br>Site visits in a prison near Tbilisi and in the narcological dispensery in Batumi.	13 interviews with injection drug users	Non IDPs	Substance Use<br>Physical health	Assessment of the prevalence of injection drug use and the transmission/potention for transmission of HIV based on patterns of risky behavior, access to health services, interventions, and drug policies
2000	Internally Displaced Persons: A Socio-Economic Survey		Internal Report	International Federation of the Red Cross	Quantitative and qualitative research			Living Conditions<br>Economic Status<br>Mental and Physical Health<br>Integration/Marginalization	Analysis of employment and health status, income, IDP registration, demographics, housing conditions and solutions, education, and nutrition
2001	Risk for tuberculosis infection among internally displaced persons in the Republic of Georgia	Weinstock, D.M, Hahn, O., Wittkamp, M. Sepkowitz, K.A. Khechinashvili, G. Blumberg, H.M	Journal Publication	International Union Against Tuberculosis and Lung Disease	Voluntary participation Tuberculin skin test and symptom questionnaire	988 Participants out of 4,000 potential candidates	IDPs living in 8 refugee hostels	Physical Health	Burden of tuberculosis among IDPs living in compact settlements
2001	Profile: Georgia	Thomas Buck, Alice L. Morton, Susan Allen Nan, and Feride Zurikashvili	Book Chapter	Women and Civil War: Impact, Organizations, and Action	Literature Review			Living Conditions<br>Economic Status<br>Integration/Marginalization<br>Mental Health	The impact of displacement on women in terms of socio-economic status, integration,

									and mental health
2002	Poverty and Vulnerability Among Internally Displaced Persons in Georgia: An Update of Their Current Status and Circumstances	Larry Dershem, Nana Gurgenidze, Steven Holtzman	Internal Report	Save the Children The World Bank	Literature Review			Living Conditions Economic Status Mental and Physical Health Integration/Marginalization Nutrition	Analysis of vulnerabilities of IDPs compared to the general population with respect to education, nutrition, mental and physical health, social capital, quality of life, housing, and dependency on social services. Study also examined poverty among IDPs (income, unemployment, food security, and business) as well as the distribution of benefits of IDP welfare benefits

2004	Profile of Internal Displacement Georgia: Compilation of the information available in the Global IDP Database of the Norwegian Refugee Council		Internal Report	Norwegian Refugee Council Global IDP Project	Literature Review		Living Conditions Economic Status Mental and Physical Health Integration/Marginalization Conflict Background	Review of publications between 1990-2004 that cover patterns of displacement, background of conflicts, territorial mobility and security, health status, access to healthcare, provision of basic needs (shelter, durable goods, food), access to education, public participation, civic engagement, psychosocial dependence, family issues, registration, patterns of resettlement and return, national and international response
2004	Living in Limbo: Conflict-Induced Displacement in Europe and Central Asia	Steven B. Holtzman and Taies Nezam	Internal Report	The World Bank	Literature Review		Living Conditions Economic status Mental and Physical Health	Review of employment status, income, livelihood opportunities, health status, demographics, material well-being, settlement patterns and housing solutions, and the social integration of IDPs in various countries

2004	Perceptions and Reality of Access to Healthcare Services among Internally Displaced Women Living in Public Facilities in Tbilisi, Georgia	Tea Collins	Doctoral Dissertation	School of Public Health and Health Services of the George Washingtion University	Literature Review<br><br>Focus group discussions	60 focus group participants	Internally displaced women living in public facilities in Tbilisi	Healthcare utilization	Perceptions of eligible healthcare services for IDPs among IDPs as well as utilization among women in collective centers
2005	Social Capital and Employment Opportunities Among Internally Displaced Persons (IDPs) in Georgia	Gocha Tskitishvili, Larry Dershem, Vano Kechakmadze	Internal Report	The World Bank	Focus group discussions and in-depth interviews<br><br>Random selection of survey households by last birthday protocol	8 FGD<br><br>20 in-depth interviews<br><br>1,003 IDPs surveyed<br><br>997 general population surveyed	FGDs with IDPs and GP in Tbilisi and Zugdidi<br><br>In-depth interviews with IDPs employed in formal or informal sector in Tbilisi, Kutaisi, and Zugdidi<br><br>Survey sample included IDPs in collective centers in Tbilisi, Kutaisi, and Zugdidi, and GP in adjacent local communities	Economic status	Analysis of barriers to employment, employment status, participation in formal and informal sectors of the labor market, comparative analysis to general population, and income from employment
2006	High prevalence of hepatitis C virus but not HIV co-infection among patients with tuberculosis in Georgia	D. C. Richards, T. Mikiashvili, J. J. Parris, E. V. Kourbatova, J. C. E. Wilson, N. Shubladze, T. Tsertvadze, G. Khechinashvili, C.	Journal Publication	International Union Against Tuberculosis and Lung Disease	Questionnaire and serologic testing	272 Patients	>18 years of age receiving treatment at in-pateitn TB hospital in Tbilisi, Poti, Batumi, and Abastuman<br><br>IDPs and non-IDPs	Physical Health	Assessment of co-morbidity of TB with HIV and Hepatitis C. Participants included IDPs

		del Rio, H. M. Blumberg							
2008	Protection of Internally Displaced Persons in Georgia: A Gap Analysis		Internal Report	United Nations High Commissioner for Refugees	Literature Review			Living Conditions<br>Economic Status<br>Mental and Physical Health<br>Integration/Marginalization<br>Conflict Background<br>Gender based violence	Review of displacement context, legal framework for institutional protection of IDPs, IDP registration and legal status in Georgia, participation in public civic life and internal community structures, access to health care, educational resources, health status, economic status (employment, income, poverty), durable housing solutions, and barriers to employment security of IDPs from sexual/gender-based violence, child recruitment ,
2008	Maternal Health in Post-Soviet Georgia	Tamar Dagargulia & Medo Badashvili	Journal Publication	Marriage & Family Review	Literature Review			Maternal Health	Overview of maternal health, morbidity and mortality, including the status of IDP women

2008	Women's Sexual and ReproductiveHealth in Post-Socialist Georgia: Does Internal Displacement Matter?	Khatuna Doliashvili and Cynthia J.Buckley	Journal Publication	International Family Planning Perspectives	Retrospective analysis of 1999 Georgia Reproductive Health Survey	268 IDP 5,435 Non-IDP	IDP and non-IDP sexually experienced women	Sexual and Reproductive Health	Assessment of morbidity and mortality of STIs and reproductive health among IDP women
2009	Food security, child nutrition, and agricultural livelihoods of conflict-affected persons in Georgia		Internal Report	Joint FAO/UNICEF/WFP	Physical examination of random sample from three clusters divided into two groups<br><br>Randomized household interview<br><br>Nonrandomized extensive assessment and semi-structured interview	Physical examination: 1,888 (0-23 months old) 2,014 (24-59 months old)<br><br>Household Interview: 100 households in 20 villages<br><br>Extensive assessment: 3 settlements and 7 villages	IDPs in collective centers, resettlement areas, returnees to villages adjacent boundary zone with South Ossetia<br><br>Physical examination of IDPs aged 0-59 months	Economic status Living Conditions Physical Health Nutrition	Study on child nutritional status, food security and livelihoods in collective centers, status of agricultural participation, barriers to agricultural enterprise, socio-economic status of IDPs in resettled areas
2009	Assessment of IDP Livelihoods in Georgia: Facts and Policies		Internal Report	United Nations High Commissioner for Refugees	Literature Review			Living Conditions Economic Status Mental and Physical Health Integration/Marginalization Conflict Background Gender based violence	Review of displacement context, housing status, and socio-economic challenges

2009	IDPs in Georgia still need attention A profile of the internal displacement situation		Internal Report	Internal Displacement Monitoring Centre	Literature Review			Living Conditions<br>Economic status	Background of conflicts, current displacement context, IDP population dynamics, physical security, IDP status and territorial mobility, access to food, water, housing, medical care, and sanitation, economic and livelihood opportunities, process of providing durable accomodations, IDP registration, protection of special IDP groups (children, women, indigenous peoples, etc.), national and international humanitarian response
2009	Post-Conflict Displacement: Isolation and Integration in Georgia	Beth Mitchneck, Olga V. Mayorova, Joanna Regulska	Internal Report	Annals of the Association of American Geographers	Face-to-face interviews<br><br>Use of structured questions, narrative questions, a formal social network analysis, and a daily path analysis.	120 IDPs<br><br>60 Non-IDPs	Male and female IDPs living in collective centers in Tbilisi, Kutaisi, and Zugdidi, as well as the adjacent local population	Integration/Marginalization<br>Socialization	Study on the social integration of IDPs, including an analysis of the scale, scope, and diveristy of social networks of IDPs living in collective centers compared to the host population post-2008

2009	Baseline Survey of the IDP Settlements and their Neighbouring Communities in Kvemo Kartli and Shida Kartli		Internal Report	GeoWel Research Caucasus Research Resource Center CARE International	Household survey	470 IDP Household 637 Non-IDP Household	IDPs and Non-IDPs in Shida Kartli and Kvemo Kartli	Living Conditions Economic Status Integration/Marginalization	Assessment of the living conditions, poverty levels, income, property, and socialization patterns of IDPs in settlement ares
2010	In the Waiting Room: Internally Displaced People in Georgia		Internal Report	Amnesty International	Literature Review<br><br>Interviews with civil society activists, representatives of displaced communities, state officials, representatives of international organizations, health professionals dealing with displaced populations and a wide variety of displaced persons			Living Conditions Economic Status Mental and Physical Health Integration/Marginalization Conflict Background	Review of conflicts, displacement, national framework, and human rights concerns, namely: IDP status and registration, right to housing, employment, social security, health, integration, and accountability
2010	Gender Assessment	Elizabeth Duban	Internal Report	USAID DevTech Systems	Desk review of USAID materials, revisions to the Automated Directives System on gender integration and a range of reports, briefings and articles from other donor agencies, international organizations, non-governmental organizations and news sources.<br><br>Relied on consultant meetings				Needs assessment of IDP women

2010	High Mortality among Patients with Positive Blood Cultures at a Children's Hospital in Tbilisi, Georgia	Jami Schaffner, Sopio Chochua, Ekaterina V. Kourbatova, Maribel Barragan, Yun F Wang, Henry M Blumberg, Carlos del Rio, H. Kenneth Walker, and Michael K. Leonard,	Journal Publication	Journal of Infection in Developing Countries	Retrospective analysis of Children's Hospital patient data	237 Non-IDP Children<br>62 IDP Children	All patients with at least one positive blood culture	Physical Health	Retrospective review of infant morbidity/ mortality with significant portion of participants reporting IDP status
2011	Accountability in Aid Delivery - The Renovation of Collective Centers for Georgia's Internally Displaced Persons		Internal Report	Transparency International Georgia	Randomized visitation of collective centers and in-person interviews	22 collective center buildings<br>90 households	Collective centers in Samegrelo, Imereti, and Adjara	Living Conditions	Review of the renovation and construction process for new and rehabilitated collective centers<br>Legislative framework of housing initiatives for IDPs post 2008<br>Study on the quality and scope of collective center renovations and new constructions
2011	Displacement in Georgia: IDP attitudes to conflict, return and justice	Magdalena Frichova Grono	Internal Report	Conciliation Resources	Summary and analysis of "IDPs in Georgia" survey			Living Conditions<br>Economic Status<br>Integration/Marginalization	Review of survey results on socio-economic and housing conditions of IDPs, as well as perceptions social integration and the prospect of returning to homes lost in occupied territories

2011	Part protracted, part progress: Durable solutions for IDPs through local integration in Georgia	Nadine Walicki	Internal Report	The Brookings Institution London School of Economics	Literature Review			Living Conditions Economic Status Integration/Marginalization	Review of long-term strategies for durabl accomodations for IDPs and update on progress made
2011	Prevalence of HIV among injection drug users in Georgia	Ivdity Chikovani, Ketevan Goguadze, Sudit Ranade, Mollie Wertlieb, Natia Rukhadze, George Gotsadze	Journal Publication	Journal of the International AIDS Society	Cross-sectional bio-behavioral survey Respondent-driven sampling Face-to-Face Interviews	1127 IDU	>18 years of age injection drug users, general population, living in Tbilisi, Gori, Telavi, Zugdidi, and Batumi	Substance Use Physical health	Prevalence of HIV among IDUs and evaluation of pathways of transmission and risk factors
2012	The Impact of Socially Ir/responsible Resettlement on the Livelihoods of Internally Displaced Persons in Georgia	Nato Kurshitashvili	Journal Publication	Refugee Survey Quarterly	Literature Review			Living Conditions Economic Status	Insufficiencies of post-2008 resettlement process in providing IDPs adequate and durable living accomodations Economic impact on resettling urban IDPs to rural areas Effect of resettlement on agricultural IDPs
2012	Partial progress towards durable solutions for IDPs		Internal Report	Internal Displacement Monitoring Centre	Literature Review			Living Conditions Economic Status	Review of progress on providing durable accomodations to IDPs, displacement context, background of conflict, employment and livelihood, terrtorial mobility, return, education, housing, and national

									response
2012	Internally Displaced Persons in the Georgian Labour Market	Mirian Tukhashvili, Murman Tsartsidze, Tsiuri Antadze, Natela Latsabidze, Mzia Shelia, Mamuka Toria	Internal Report	Ivane Javakhshvili Tbilisi State University Center for Migration Studies	Face-to-face interview	202 IDPs	Male and female unemployed IDPs from Abkhazia and South Ossetia living in collective centers in Tbilisi, Zugdidi, and Gori	Economic status	Unemployment, barriers to employment, comparative analysis of labour force participation between IDPs and general population, policy recommendations
2012	Aging in Displacement: Assessing the Health Status of Displaced Older Adults in the Republic of Georgia	George Rebok, Judy Bass, Paul Perrin, Namrita Singh, Nino Paichadze, Holly Schuh, Tamar Abashidze, Irina Zhvania, Tamar Makharadze, Maia Maisuradze, Ekaterine, Pirtskhalava.	Internal Report	Johns Hopkins Bloomberg School of Public Health<br>US Department of State Bureau of Population, Refugees and Migration	Free-list and key informant inerview<br><br>Household survey	75 Free-list interview<br>45 Key informant interview<br>899 Household interviews	Survey sample included OCL and NCL IDPs in collective centers and private dwellings aged 65 years and older	Mental Health	Evaluation of mental illness (PTSD, GAD, depression) of elderly displaced persons, dissociated by gender, dwelling type, and caseload.

2012	Insomnia in a Displaced Population is Related to War Associated Remembered Stress	Tamar Basishvili, Marine Eliozishvili, Lia Maisuradze, Nani Lortkipanidze, Nargiz Nachkebia, Tengiz Oniani, Irma Gvilia, Nato Darchia	Journal Publication	Stress and Health	Sleep interviews and medical examinations by sleep specialists and physicians<br><br>Face-to-face interview<br><br>Randomly selected participants	105 IDPs	IDPs from Sukhumi (Abkhazia) living in five different collective centers in Tbilisi<br><br>Participants aged 20-60 years old	Mental Health	Assessment of insomnia in IDPs and factors associated with insomnia: demographic, socioeconomic, traumatic events
2012	Methods of investigation of social protection and Nutrition Problem in IDPs of Georgia	N.Gokieli, D.RaminaSvili, Sh.Zarnadze, I. Zarnadze, L.Lomtadze, D.kitovani, M.Kajrishvili	Journal Publication	Стратегические вопросы мировой науки	Cross sectional study Household survey and interview	450 IDPs	IDP	Nutrition	Implementation of a new study of nutritional status and social protection of IDPs
2012	Behavioral Surveillance Survey with biomarker component on HIV/AIDS in high risk groups, identifying the number of injective drug users, operations survey		Internal Report	Curatio International Foundation<br>Bemoni Public Union<br>The Global Fund	Cross-sectional bio-behavioral survey<br>Respondent-driven sampling<br>Face-to-Face Interviews			Substance Use<br>Physical health	Prevalence of HIV among IDUs, female sex workers, prison inmates, and men who have sex with men
2012	HIV risk and prevention behaviours among People Who Inject Drugs in six cities of Georgia Bio-behaviora	Ivdity Chikovani, Natia Shengelia, Nino Chkhaidze, Tamara Sirbiladze, Lela Tavzarashvili	Internal Report	Curatio International Foundation<br>Bemoni Public Union<br>The Global Fund<br>National Center for Disease Control and Public Health	Cross-sectional bio-behavioral survey<br>Respondent-driven sampling<br>Face-to-Face Interviews	1740 IDU	>18 years of age injection drug users, general population, living in Tbilisi, Gori, Telavi, Zugdidi, Batumi, and Kutaisi	Substance Use<br>Physical health	Prevalence of HIV among IDUs and assessment of protective and risky behaviors and utilization of harm reduction resources

	l surveillance survey in Tbilisi, Batumi, Zugdidi, Telavi, Gori, Kutaisi							
2013	Supporting the Livelihoods of Internally Displaced Persons in Georgia: A Review of Current Practices and Lessons Learned		Internal Report	The World Bank Social Development Europe and Central Asia	Desk study and research<br><br>individual interviews with livelihood providers, relevant government ministries and departments, UN agencies, donors, beneficiaries, and other stakeholders;<br><br>Field visits to livelihood programs where meetings were held with local authorities (beneficiary communities and individuals were interviewed via individual meetings and community-based focus groups);<br><br>Peer review feedback of the draft report via email and face-to-face interaction		Economic status<br>Living Conditions<br>Integration/Marginalization	Review of conflict and displacement, the housing and socio-economic status of IDPs, and barriers to sustainable livelihoods for IDPs, namely: access to land and financial institutions, social capital, skills, and dependency on aid
2013	Asylum Seekers, Refugees and Internally Displaced Persons (IDPs) in Georgia: the challenges of social cohesion	Natia Chelidze	Internal Report	Consortium for Applied Research on International Migration	Literature Review		Living Conditions<br>Economic status	Review of displacement context, housing status, and socio-economic challenges

2013	Economic and Social Vulnerability in Georgia	Franziska Gassmann, George Berulava, Michael Tokmazishvili	Internal Report	United Nations Development Programme in Georgia	Analysis of data from Household Budget Survey (GEOSTAT)<br><br>In-depth interviews and focus group discussion with representatives of target groups	Survey analysis: 4,301 households	IDP households<br><br>Households with disabled persons<br><br>Households in high mountain areas	Living Conditions<br>Economic Status<br>Integration/Marginalization	Review of displacement context, housing status, employment, income, economic vulnerabilities
2013	Coping with marginality and exclusion: can refugees communities successfully integrate into mainstream urban societies in Georgia?	Joseph Salukvadze , Revaz Gachechiladze, David Gogishvili, David Sichinava, Anna Javashvili, Ketevan Tugushi, Mariam Bregvadze	Internal Report	Tbilisi State University Faculty of Social & Political Studies Department of Human Geography Academic Swiss Caucasus Net	Face-to-face semi-structured interviews selected by quota sampling of major gender-age groups<br><br>Analysis of government documents, international organization reports, and academic resources	900	9 urban settlements with highest concentration of IDPs in collective centers and one rural settlement<br><br>Aged 18 years and over	Living Conditions<br>Economic Status<br>Integration/Marginalization	Study on the socio-economic vulnerabilities of IDPs in collective centers<br><br>Effect of collective centers on social integration and marginalization
2013	Tobacco Use and Nicotine Dependence among Conflict-Affected Men in the Republic of Georgia	Bayard Roberts, Ivdity Chikovani , Nino Makhashvili , Vikram Patel, Martin McKee	Journal Publication	International Journal of Environmental Research and Public Health	Cross sectional study<br>Multi-stage random sampling with stratification by region and displacement status	1200 Old Caseload<br>1200 New Caseload<br>1200 Returnee		Substance Use<br>Mental health	Analysis of patterns of tobacco use and nicotine dependence among IDPs, as well as risk factors to use
2014	Population Situation Analysis Georgia	Mr. Ralph Hakkert, Ms. Gulnara Kadyrkulova, Mrs. Nata Avaliani, Mr. Eduard Jongstra, Mr. Lasha Labadze, Ms. Maka Chitanava, Ms. Nino Doghonadze;	Internal Report	United Nations Population Fund	Based on the methodology for PSAs developed by the Population and Development Branch of the Technical Division of UNFPA. Contains some main elements of the PSA format, but some elements are missing<br><br>Unclear methodology			Physical Health<br>Economic status	Broad review of population dynamics, sexual and reproductive health, housing structure, economic status, and challenges/opportunities facing data collection and fertility for the general population, with reference to the status of IDPs throughout

2014	The Multiple Geographies of Internal Displacement: The Case of Georgia	Peter Kabachnik, Beth Mitchneck, Olga V. Mayorova, Joanna Regulska	Journal Publication	Refugee Survey Quarterly	Face-to-face interviews in four surveys (structured question, narrative question, institutional interview, combination of structured and narrative questions)	Survey 1: 118 IDPs 62 Local population<br><br>Survey 2: 37 government, NGO, and IGO officials<br><br>Survey 3: 32 IDPs<br><br>Survey 4: 60 IDPs	Survey 1: IDPs in collective centers and private dwellings, working age, men and women, living in Tbilisi, Zugdidi, and Kutaisi<br><br>Survey 3: IDPs living in Tbilisi<br><br>Survey 4: IDPs from South Ossetia in four different settlements who were resettled almost immediately from collective centres to new settlements	Living Conditions Integration/Marginalization	Review of frameworks for understanding the experience of forced migrant populations. Emphasis on the need to present data on internally displaced persons in a categorical manner that considers geographical perspectives on the socio-economic circumstances of IDPs rather than broad generalizations that may fail to disclose hidden experiences
2014	Individual and Community Level Risk-Factors for Alcohol Use Disorder among Conflict-Affected Persons in Georgia	Bayard Roberts, Adrianna Murphy, Ivdity Chikovani, Nino Makhashvili, Vikram Patel, Martin McKee	Journal Publication	PLOS One	Cross sectional study Multi-stage random sampling with stratification by region and displacement status	1200 Old Caseload 1200 New Caseload 1200 Returnee		Substance Use Mental health	Analysis of patterns of alcohol use among IDPs and risk factors
2014	Mental Disorders and Their Association With Disability Among Internally Displaced Persons and Returnees in Georgia	Nino Makhashvili, Ivdity Chikovani, Martin McKee, Jonathan Bisson, Vikram Patel, Bayard Roberts	Journal Publication	Journal of Traumatic Stress	Cross sectional study Multi-stage random sampling with stratification by region and displacement status	1193 Old Caseload 996 New Caseload 836 Returnee	OCL, NCL, and Returnee IDPs	Mental Health	Assessment of mental illness among IDPs and risk factors, including demographics (age, gender), disability, and socio-economic status

									(household economic condition, housing conditions, education, relationship status).
2014	Comparative Study of Psychological Well-Being and Posttraumatic Growth Indicators in IDP and Non-IDP Citizens of Georgia	Lili Khechuashvili	Journal Publication	GESJ: Education Science and Psychology	Simple probability sampling procedure Face-to-face interview	212 Inventory adaptation 589 Main research	IDPs and non-IDPs aged 25-50	Mental Health	Comparitive assessment of positive outcome reported by traumatized displaced persons and non-displaced population
2014	Internally Displaced Person in Georgia: Gaps in Law and Practice	Nino Mchedlishvili	Masters Thesis	Faculty of Law Lund University	Literature Review			Living Conditions Economic status	Background of conflicts, legal framework for the care and integration of IDPs, and assessment of IDP socio-economic and living conditions with reference to legal commitments
2015	Intentions Survey on Durable Solutions: Voices of Internally Displaced Persons in Georgia		Internal Report	Institute of Social Studies and Analysis United Nations High Commissioner for Refugees	Focus group discussion in preparation for field work<br><br>Face-to-face survey interviews	2,001	NCL IDPs from South Ossetia and Abkhazia living in collective centers (including privatized, newly built, rehabilitated and non-rehabilitated	Living Conditions Economic Status Integration/Marginalization	Study on the socio-economic status of IDPs, perceptions on durable solutions, status of living accomodations, process of resettlement, integration in host communitie

							collective centers), cottage settlements, and private accommodation		s, and the perceptions and scope of voluntary return to occupied territories
2015	Georgia Urban Strategy Priority Area III: Housing	Ahmed A. R. Eiweida, Ashna Mathema, Joseph Salukvadze, Max Budovitch, Elena Darjania, Vladimer Vardosanidze	Internal Report	Ministry of Economy and Sustainable Development of Georgia<br>The World Bank	Desk review<br><br>Field visits and interviews with residents and relevant stakeholders in Tbilisi			Living Conditions<br>Economic status	Review and analysis of the housing market (affordability, supply and demand, housing stock and their quality) as well as the legal framework of housing initiatives aimed at vulnerable populations and the housing status of IDPs
2015	Urban dimensions of internal displacement in Georgia: The phenomenon and the housing policy	David Gogishvili	Conference Paper	RC21 International Conference on “The Ideal City: between myth and reality. Representations, policies, contradictions and challenges for tomorrow's urban life”	Literature Review<br><br>Review of survey conducted between 2011-13<br><br>In-person interview and survey questionnaire			Living Conditions<br>Economic Status<br>Integration/Marginalization	Review of survey results conducted in 2013 on the socio-economic vulnerabilities of IDPs in collective centers<br><br>Effect of collective centers on social integration and marginalization

2015	Patterns of somatic distress among conflict-affected persons in the Republic of Georgia	Ruben Moreno Comellas, Nino Makhashvili, Ivdity Chikovani, Vikram Patel, Martin McKee, Jonathan Bisson, Bayard Roberts	Journal Publication	Journal of Psychosomatic Research	Cross sectional study Multi-stage random sampling with stratification by region and displacement status	1200 Old Caseload 1200 New Caseload 1200 Returnee	OCL, NCL, and Returnee IDPs	Mental Health	Assessment of somatic distress among IDPs and its association with demographic (age, gender), socio-economic status, and other mental illness (PTSD, anxiety)
2015	Health Service Utilization for Mental, Behavioural and Emotional Problems among Conflict-Affected Population in Georgia: A Cross-Sectional Study	Ivdity Chikovani, Nino Makhashvili , George Gotsadze , Vikram Patel, Martin McKee, Maia Uchaneishvili, Natia Rukhadze, Bayard Roberts	Journal Publication	PLOS One	Cross sectional study Multi-stage random sampling with stratification by region and displacement status	1200 Old Caseload 1200 New Caseload 1200 Returnee	OCL, NCL, and Returnee IDPs	Mental Health Healthcare utilization	Assessment of healthcare utilization among IDPs and the association with demographics, socio-economic status, and mental illness
2015	Protracted Displacement in Georgia: Structural Vulnerability and "Existing not Living"	Erin Koch	Journal Publication	Human Organization	Literature Review Semi-structured interview and participant observation	55 Individuals in 11 collective centers	IDPs in collective centers in or around Zugdidi Government officials and NGO workers	Living Conditions Economic Status Integration/Marginalization Mental Health	Research in collective centers, includes broad review of conflict and the status of IDPs in Georgia
2015	Health Service Utilization, Expenditures and Health Status among IDP Population in Georgia		Internal Report	Elrha	Household survey of collective centers Multistage sampling approach	1,319 Households 4,359 Household members	IDPs living in collective centers in Tbilisi, Imereti, Kvemo Kartli, Adjara, Samegrelo-Zemo Svaneti, Shida Kartli, and Mtskheta-Mtianeti	Physical and Mental Health Healthcare utilization	Self-reported health status (chronic illness, hospital visitations) of IDPs in collective centers and utilization of healthcare system/benefits to address health needs

2015	An assessment of socio-economic integration and livelihood needs of IDPs in Georgia	Sean Loughna	Internal Report	Analysis and Consulting Team<br>European Union<br>Hulla & Co. Human Dynamics KG	Household survey<br>Personal interview, key informant interview, focus group discussion	1996 IDP Households<br>514 Non-IDP Households	IDPs and non-IDPs in Imereti, Adjara, Samegrelo, Kvemo Kartlie, Shida Kartli, Mtskheta Mtianeti, and Tbilisi	Living Conditions<br>Economic Status<br>Mental and Physical Health<br>Integration/Marginalization	Assessment of living conditions, economic status (employment, poverty), mental and physical health indicators, and socialization patterns among IDPs
2015	National Survey on Substance Use in the General Population in Georgia	Irma Kirtadze, David Otiashvili. Mzia Tabatadze	Internal Report	USAID<br>Addiction Research Center Alternative Georgia<br>Czech Development Agency	Multi-stage sampling, survey questionnaire, face-to-face interview	3,228 Households<br>4,805 Individual respondents	General population between 18 and 64 years of age	Substance Use	Estimate of the IDU population size in Georgia
2015	HIV risk and prevention behaviors among People Who Inject Drugs in seven cities of Georgia<br><br>Bio-Behavioral Surveillance Survey in seven cities of Georgia	Ivdity Chikovani, Natia Shengelia, Lela Sulaberidze, Tamara Sirbiladze, Lela Tavzarashvili	Internal Report	Curatio International Foundation<br>Bemoni Public Union<br>The Global Fund<br>Infectious Disease, AIDS and Clinical Immunology Research Center	Cross-sectional bio-behavioral survey<br>Respondent-driven sampling<br>Face-to-Face Interviews	2050 IDU	>18 years of age injection drug user, general population, living in Tbilisi, Gori, Telavi, Zugdidi, Batumi, Kutaisi and Rustavi.	Substance Use<br>Physical health	Prevalence of HIV among IDUs and assessment of protective and risky behaviors and utilization of harm reduction resources

2015	Population Size Estimation of People who Inject Drugs in Georgia 2014	Tamar Sirbiladze, Lela Tavzarashvili, Ivdity Chikovani, Natia Shengelia, Lela Sulaberidze .	Internal Report	Curatio International Foundation<br>Bemoni Public Union<br>The Global Fund	Population-based surveys<br><br>Casefinding studies<br><br>Capture-recapture estimates<br><br>Multiplier techniques<br><br>Nomination techniques<br><br>Synthetic estimates, based on social or demographic variables assumed to correlate with drug prevalence	1951 IDU	>18 years of age injection drug user, general population, living in Tbilisi, Gori, Telavi, Zugdidi, Batumi, Kutaisi and Rustavi.	Substance Use	Estimate of the IDU population size in Georgia
2016	Transitioning from Needs Based Assistance for IDPs: A Poverty and Social Impact Analysis		Internal Report	The World Bank	Desk review of Georgia's laws, policies, and institutions for IDP assistance compared to international good practice<br><br>Analysis of survey data from Integrated Household Survey (2011-13; National Statistics Office of Georgia), UNHCR Intentions Survey (2015), and UNDP Household Survey (2011)<br><br>Focus group discussions with IDPs and non-IDPs in urban and rural areas in Tbilisi, Kakheti, Samegrelo, and Shida Kartli<br><br>Key informant interview with policy makers, public institution representatives at national and local levels, nongovernment	24 FGD<br>10 KII<br>5 Ethnographic case studies		Living Conditions<br>Economic Status<br>Integration/Marginalization	Review of legal framework for interventions aimed at IDP issues, socio-economic status of IDPs, and welfare benefits for IDPs, as well as policy recommendations

					al organizations, IDP leaders, and international development partners.<br><br>Ethnographic case studies on IDPs in collective centers, private accomodation, dependent on social assistance, disabled, and residing alone				
2016	Significant Improvements Are Needed in HIV Care Continuum to Meet 90-90-90 Targets in Georgia	Nikoloz Chkhartishvili, Otar Chokoshvili, Natia Dvali, Akaki Abutidze, Lali Sharvadze, Tengiz Tsertsvadze	Journal Publication	Journal of the International Association of Providers of AIDS Care	Retrospective analysis on patient data from AIDS Health Information System			Physical Health	Evaluation of progress towards nationwide 90-90-90 targets for HIV
2016	“Finding a way out”: Case histories of mental health care-seeking and	Namrita S. Singh, Nino Jakhaia, Nino Amonashvili, Peter J. Winch	Journal Publication	Transcultural Psychiatry	In-depth semi-structured interviews Chain sampling selection approach	9 IDPs	Individuals who accessed mental health care from formal services<br><br>IDPs	Mental Health	Assessment of in-depth IDP case studies derived from earlier study

	recovery among long-term internally displaced persons in Georgia						living in collective centers in Zugdidi, Poti, and Tbilisi		
2017	Gender Based Violence in Georgia: Links Among Conflict, Economic Opportunities and Services		Internal Report	World Bank Group Social, Urban, Rural & Resilience State and Peacebuilding Fund	Desk review of institutions engaged in GBV, GBV-related policies, and background note on LGBT people<br><br>Randomized stratified quantitative survey<br><br>Focus group discussions (FGD), purposive sampling<br><br>Key informant interviews, purposive and snowball sampling	3,014 survey interviews<br><br>35 FGD (280 participants)<br><br>10 in-depth informant interviews	Survey sample: Male and female IDPs, NAPs, and ALPs<br><br>Focus groups: Male and female IDPs, NAPs, and ALPs, young and elderly women, LGBT people, GBV service practicioners<br><br>Key informants: Government officials, academics, civil society organization members, international organization officials, thematic and regional specialists	Economic status Gender based violence	Analysis of the interplay between conflict/displacement, livelihood and socioeconomic opportunities, and the current needs of conflict-affected and general populations on gender based violence
2017	Coping strategies and mental health outcomes of conflict-affected persons in the Republic of Georgia	L. Saxon, N. Makhashvili, I. Chikovani, M. Seguin, M. McKee, V. Patel, J. Bisson, B. Roberts	Journal Publication	Epidemiology and Psychiatric Sciences	Cross sectional study Multi-stage random sampling with stratification by region and displacement status	1200 Old Caseload 1200 New Caseload 1200 Returnee	OCL, NCL, and Returnee IDPs	Mental Health	Analysis of coping strategies used by IDPs with mental disorders, with comparative analysis by demographic, illness, and socio-economic

										characteristics
2017	Late presentation of HIV infection in the country of Georgia: 2012-2015	Nikoloz Chkhartishvili, Otar Chokoshvili, Natalia Bolokadze, Maya Tsintsadze, Lali Sharvadze, Pati Gabunia, Natia Dvali, Akaki Abutidze, Tengiz Tsertsvadze	Journal Publication	PLOS One	Retrospective analysis on patient data from AIDS Health Information System	2267 Individuals	General Population	Physical Health	Evaluation of progress towards nationwide 90-90-90 targets for HIV
2017	Progress Toward Achieving the UNAIDS 90-90-90 Goals in HIV Care From Diagnosis to Durable Viral Suppression in the Country of Georgia	Nikoloz Chkhartishvili, Otar Chokoshvili, Akaki Abutidze, Natia Dvali, Carlos del Rio, and Tengiz Tsertsvadze	Journal Publication	AIDS Research and Human Retroviruses	Retrospective analysis on patient data from AIDS Health Information System	1,931 Individuals	General Population	Physical Health	Evaluation of progress towards nationwide 90-90-90 targets for HIV
2017	HIV risk and prevention behaviors among People Who Inject Drugs in seven cities of Georgia<br><br>Bio-Behavioral Surveillance Survey in	Ivdity Chikovani, Natia Shengelia, Lela Sulaberidze, Tamara Sirbiladze, Lela Tavzarashvili	Internal Report	Curatio International Foundation<br>Bemoni Public Union<br>The Global Fund<br>Infectious Disease, AIDS and Clinical Immunology Research Center	Cross-sectional bio-behavioral survey<br>Respondent-driven sampling<br>Face-to-Face Interviews	2037 IDU	>18 years of age injection drug user, general population, living in Tbilisi, Gori, Telavi, Zugdidi, Batumi, Kutaisi and Rustavi.	Substance Use<br>Physical health	Prevalence of HIV among IDUs and assessment of protective and risky behaviors and utilization of harm reduction resources

	seven cities of Georgia								
2018	Durable Housing Solutions for IDPs: Lessons Learnt from Georgia & Steps forward in Ukraine		Internal Report	Danish Refugee Council (DRC) Danish Demining Group (DDG)	Literature Review			Living Condition s	Legislative reform of national action plans for providing durable housing solutions for IDPs post 2008
2018	Job market outcomes for IDPs: The case of Georgia	Karine Torosyan , Norberto Pignatti, Maksym Obrizan	Journal Publication	Journal of Comparative Economics	Retrospective analysis of Integrated Household Survey data from 2004-2016	11,440 IDPs 219,473 Non-IDPs	Time Periods (2004-08, 2008-12, 2013-16)<br><br>IDPs and Non-IDPs in Tbilisi, Samegrel o/Imereti, and Kartli/Mts kheta	Economic status	Compariso n of labor force participatio n between IDPs and the general population using Integrated Household Survey data from 2004-16
2018	Identifyin g mental health problems and Idioms of distress among older adult internally displaced persons in Georgia	Namrita S.Singha, Judith Bass, Nana Sumbadz e, George Rebok, Paul Perrina, Nino Paichadz ea, W. Courtlan d Robinson	Journal Publication	Social Science & Medicine	Free-list and key informant inerview<br><br>Household survey			Mental Health	In-depth assessment of IDP case studies derivced from earlier study

2018	Barriers to mental health care utilization among internally displaced persons in the republic of Georgia: a rapid appraisal study	Adrianna Murphy, Ivdity Chikovani, Maia Uchaneishvili, Nino Makhashvili, Bayard Roberts	Journal Publication	BMC Health Services Research	Rapid appraisal methods<br><br>Literature review of policy documents and published data<br><br>Semi-structured interviews with key informants including policy makers, NGO staff, health professionals and patients identified by snowball sampling	29 Key informants		Mental Health<br>Healthcare utilization	Barriers to mental healthcare utilization
2018	A population-based tuberculosis contact investigation in the country of Georgia	D. Baliashvili, R. R. Kempker, H. M. Blumberg, G. Kuchukhidze,, T. Merabishvili, A. Aslanikashvili, M. J. Magee	Journal Publication	International Union Against Tuberculosis and Lung Disease	Interview, Tuberculin Skin Test, Retrospective Cohort Study	2812 Non-IDP<br>72 IDP	Contacts of active PTB cases diagnosed with acid-fast bacili sputum smear-positive disease	Physical Health	Evaluation of tuberculosis contact in the general population, study included a significant proportion of IDPs
2018	Georgia HIV/AIDS National Strategic Plan		Government Report	Government of Georgia	Strategic Plan			Physical Health	National strategic plan for addressing concentrated epidemic of HIV/AIDS in high-risk groups
2019	The social and spatial insularity of internally displaced persons: "neighbourhood effects" in Georgia's collective centres	David Gogishvili and Suzanne Harris-Brandts	Journal Publication	Caucasus Survey	Literature review<br><br>Review of survey conducted between 2011-13<br><br>In-person interview and survey questionnaire	794	NCL and OCL IDPs from both Abkhazia and South Ossetia living in urban collective centers in Batumi, Gori, Kutaisi, Poti, Rustavi, Tbilisi, Tskaltubo, and Zugdidi	Living Conditions<br>Economic Status<br>Socialization	Review of survey results conducted in 2013 on the socio-economic vulnerabilities of IDPs in collective centers<br>Effect of collective centers on social integration and marginalization

2019	Hepatitis C prevalence and risk factors in Georgia, 2015: setting a baseline for elimination	Liesl M. Hagan, Ana Kasradze, Stephanie J. Salyer, Amiran Gamkrelidze, Maia Alkhazashvili, Gvantsa Chanturia, Nazibrola Chitadze, Roena Sukhiashvili, Marina Shakhnazarova, Steven Russell, Curtis Blanton, Giorgi Kuchukhidze, Davit Baliashvili, Susan Hariri, Stephen Ko, Paata Imnadze, Jan Drobeniuc, Juliette Morgan & Francisco Averhoff	Journal Publication	BMC Public Health	Cross-sectional seroprevalence survey Multi-stage cluster design, stratified by geography	7,000 Participants	General Population	Physical Health	Nationwide prevalence study on hepatitis C, including analysis of risk factors
1998-2017 *	The Situation in Human Rights and Freedoms in Georgia	Public Defender (Ombudsman) of Georgia	Government Report	Government of Georgia	Examination of 6,489 applications/complaints and statistical data provided by ministries of government			Living Conditions	Housing condtions Progress updates on the process of providing durable accomodations

2004-2015 *	The Drug Situation in Georgia		Internal Report	Alternative Georgia	Literature Review		Physical Health Substance Use	Review of drug use among the general population and various sub groups, the development and availability of harm reduction/preventative resources, prevalence of injection drug use, status of drug-related infectious diseases, drug markets, and legislative framework of criminal justice reform
2008-2017 *	Healthcare Statistical Yearbook		Government Report	National Center for Disease Control and Public Health Ministry of Internally Displaced Persons From the Occupied Territories, Labour, Health and Social Affairs of Georgia	Literature Review		Physical and Mental Health	Yearly overview of morbidity and mortality of endemic diseases, including prevalence, incidence, and transmission of major infectious diseases like HIV and Hepatitis C
2013-2016 *	Ministry of Internally Displaced Persons from the Occupied Territories, Accommodation and Refugees of Georgia Report		Government Report	Ministry of Internally Displaced Persons from the Occupied Territories, Accommodation and Refugees of Georgia	Document review and in-person assessment		Living Conditions	Overview of progress made in the process of providing durable accomodations to IDPs, reported annually. Record of new construction projects, provision of rental assistance, and privatization of homes

*Reports published annually

Demographics of Georgian IDPs

There are several important sides to this statistic that should be considered: (1) IDP status is inherited and so descendants who might have lived in Georgia their entire lives are still considered to be IDPs, (2) registration for IDP status is optional and thus the figure is not entirely representative, and (3) a number of IDPs, mostly from Abkhazia, have been able to return to their homes. [1]

Roughly 53% of IDPs are women, 28% are children under the age of 18, and 13% are over 65 years old. According to the Ministry, 78% of IDPs live in urban areas primarily in Tbilisi (38%) and Samegrelo-Zemo Svaneti (32%). IDP living arrangements generally fall under three categories: collective centers (CC), private accommodation (PA), and new settlements. There are about 1,600 CCs, either renovated or non-renovated, mostly located in regions adjacent to the borders with Abkhazia and South Ossetia.[1]

Living Conditions

The condition of living spaces and dwellings in CCs present unique challenges to the IDPs living in them. Prior to 2008, these conditions were particularly impacted by the critical weakness of the Georgian state. In the decade following the first state conflict (1993), the Georgian state struggled to recover from institutional incompetence and the near-collapse of the economy. Consequentially, the state was unprepared and ill-equipped to implement durable housing initiatives to properly accommodate the influx of old-caseload IDPs. Roughly half of these IDPs were forced to utilize existing building stock provided either officially by the state or squatted. The lack of institutional support meant many of these collective centers were not durable enough for permanent housing, and plans to convert them into durable spaces were ineffectual and underfunded. [2]

Unsurprisingly, reports between 1999 and 2008 have repeatedly uncovered substandard living conditions of CCs during this period with no clear indication of improvement over time. A 2002 survey of CCs in west Georgia conducted by Save the Children revealed only 5% of IDPs claiming their CC was in good condition, contrasted with 70.5% which mentioned needing major repairs and 24.1% mentioning only minor repairs.[3] The major repairs needed were predominately concerned with basic infrastructure, such as leaking roofs, walls in disrepair, broken windows, dysfunctional sewage and electrical systems. The IRFC (2000)[4], UN OCHA (2003)[5], and UNHCR (2008)[6] [7] all report the same shortcomings, adding inadequate access to water and bathing facilities, as well as improper insulation, exposed and unsafe electric systems, and structurally compromised buildings as related issues. The IFRC (2000) and SC (2002) both also report poor hygiene in CCs: 37-40% of CC-IDPs had access to an unshared toilet compared to 70-95% of the general population nationally. Rural CCs fared even worse with only 6% of inhabitants having unshared access to toilets, and only 30% to kitchens (IFRC 2000).

Debilitating overcrowding is another widely observed issue. The IFRC (2000) and SC (2002) reported average living spaces of 7-9m^2 in CCs compared to 18-30m^2 for the general population. The prevalence of these deficiencies across all CCs is difficult to determine. According to the UN OCHA (2003), 70% of CCs did not meet minimum living standards due to various combinations of the failures described above. Following a 2005 visit to Georgia, the UNHCR Representative of the Secretary General (RSG) described the living conditions of CC-IDPs as "miserable" and "shocking", concluding that they violate the right to adequate housing. In a subsequent visit in October 2008, shortly after the second state conflict, the RSG noted the "deplorable standard" of public buildings constituting many of the CCs housing old caseload IDPs. Qualitative data from interviews with CC-IDPs poignantly reinforce these findings with descriptions of living conditions as "degraded", "extremely sub-standard". "dilapidated", and "congested".[6]

Post-2008, but prior to reforms between 2012-14, there were several additional criticisms of the resettlement process. Refurbishment works have not been carried out in most of the privatized objects and in newly built ones, the quality is indicated to be poor. No significant improvements were identified in cottage-type settlements in 2011. During the allocation process, there were instances where families with two members were given a one-room apartment, while other two-member households received two-room apartments. Some IDP households that qualified for three room apartments reported being offered newly built accommodations consisting of 1 or 2 room.[8 9] As for lack of infrastructure, the assessment of several resettlement areas in Shida-Kartli revealed that roughly 80% of the population needed to cover more than 5 km to reach the nearest wholesale market (FAO/UNICEF 2009).[10] Households lacked agricultural land plots, and in many cases these were of poor quality.[10 11 12] The privatization process was rebuked for difficulty securing loans, miscommunication of process guidelines and procedures, lack of awareness, financial exposure, and insufficient housing subsidies, especially in urban areas were opportunities for improving livelihood are more accessible for landless IDPs.[8]

Furthermore, not only were some households offered smaller accommodations than they qualified for – forcing them to choose between accepting smaller homes or splitting the family into two blocks, which automatically went against the family unity principle – but the results of post-relocation monitoring (2011) also showed that access to livelihood and employment opportunities for IDPs being resettled from Tbilisi had not improved and that local infrastructure in new regions did not give IDPs the opportunity to satisfy their basic social and economic needs.[8] For example, in many settlements, land allocated for farming or purposeful labor was located several kilometers away from farmers' cottages, creating a daily commute that is time-consuming, costly, and physically exhaustive. Moreover, new accommodations built in rural areas were meant to be occupied by new caseload IDPs to avoid disruption social networks established by old caseload IDPs; however, several cases in 2010 and 2011 revealed old caseload IDPs being evicted from their CCs and offered alternative housing in rural areas where livelihood opportunities are limited.[11] One of these instances reported in August 2010 uncovered the eviction of IDPs who occupied government buildings in Tbilisi and were resettled away from the capital. Evictees were effectively cut off from their social networks, as well as sources of income, healthcare, and education. In response, some IDPs emigrated back in search for employment and sustainable livelihoods.[11]

Initially, funding for these objectives was covered in the millions of US-Dollars pledged by international donors and later subsumed in the state budget. For instance, between 2009-11 EU-backed financing allowed for the renovation of over 7,000 apartments accommodating over 4,300 families.[13]

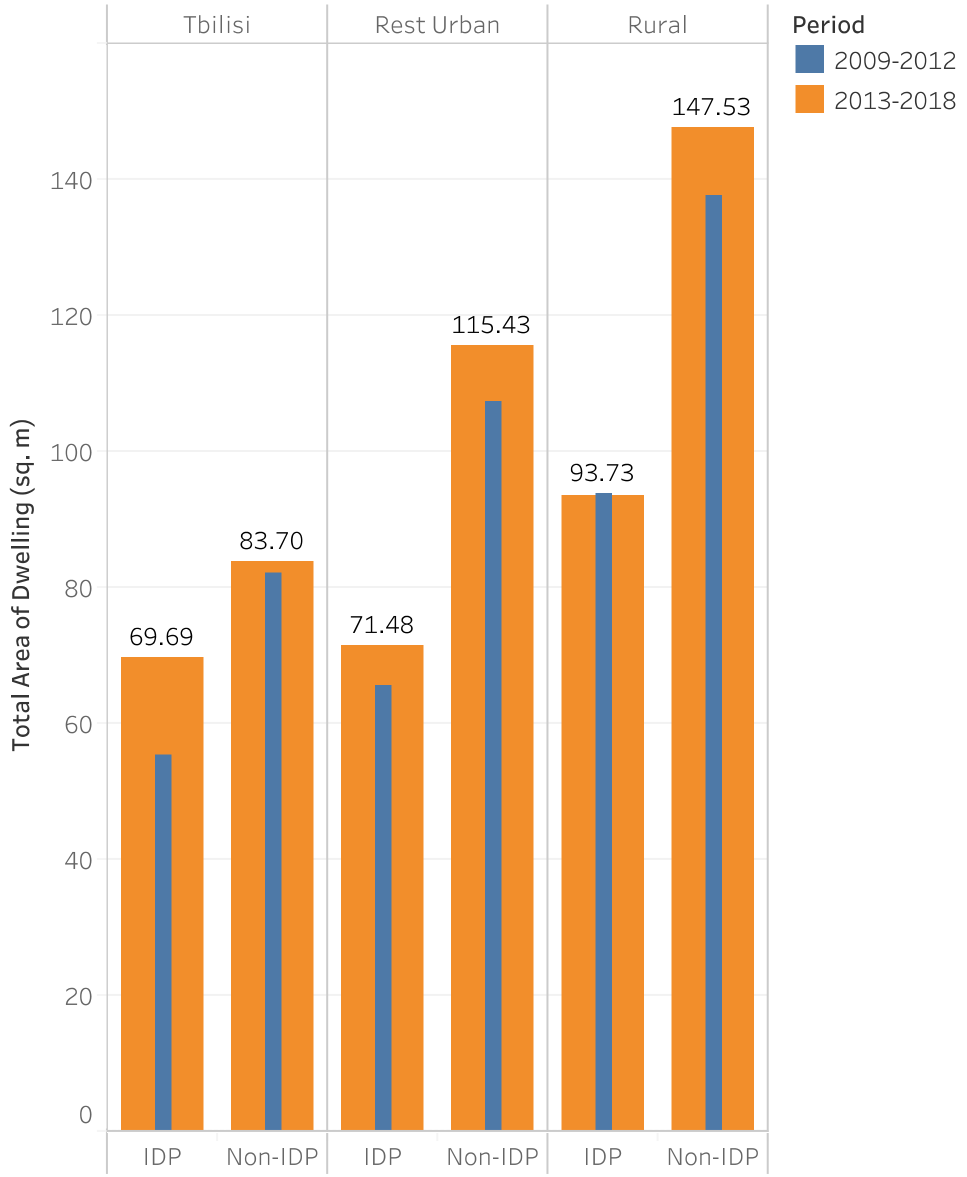

Figure 1: Total area of dwelling by square meter. Comparison of dwelling size between geographic areas, IDP status, and time periods.

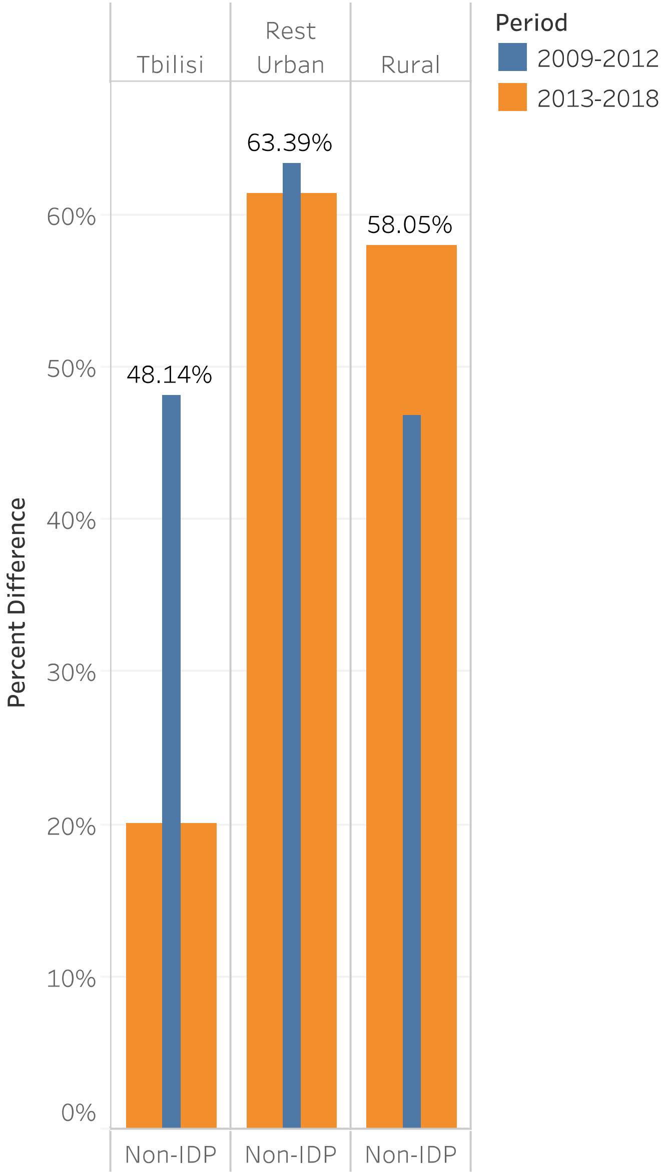

Figure 2: Percent difference of dwelling size by status. Percent difference in dwelling size between IDPs and non-IDPs across geographic areas and time periods

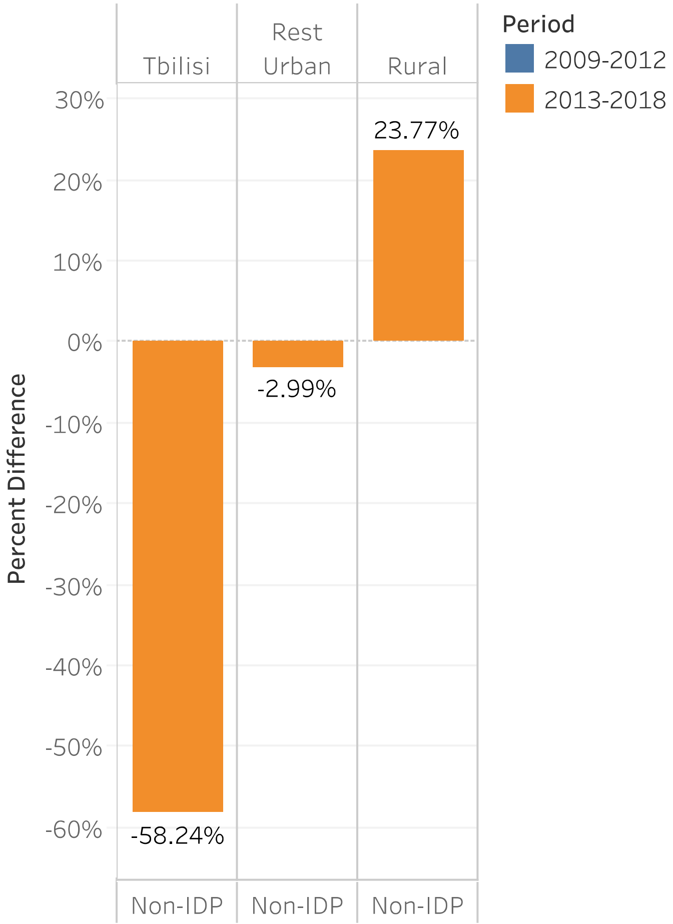

Figure 2: Percent difference in dwelling size gap over time.

According to the IHS, the total dwelling size of IDPs and non-IDPs increased overtime across all geographic regions. In both time periods, the dwelling size of IDPs were smaller than non-IDPs across all regions by 20%-63%, with the greatest consistent disparity seen in other urban areas and lowest in Tbilisi. The gap in dwelling size appears to be closing dramatically in Tbilisi (58%) and less so in other urban areas (3%), but increasing in rural areas (23%). This largely reflects relative stagnation and improvement in either IDPs or non-IDPs.

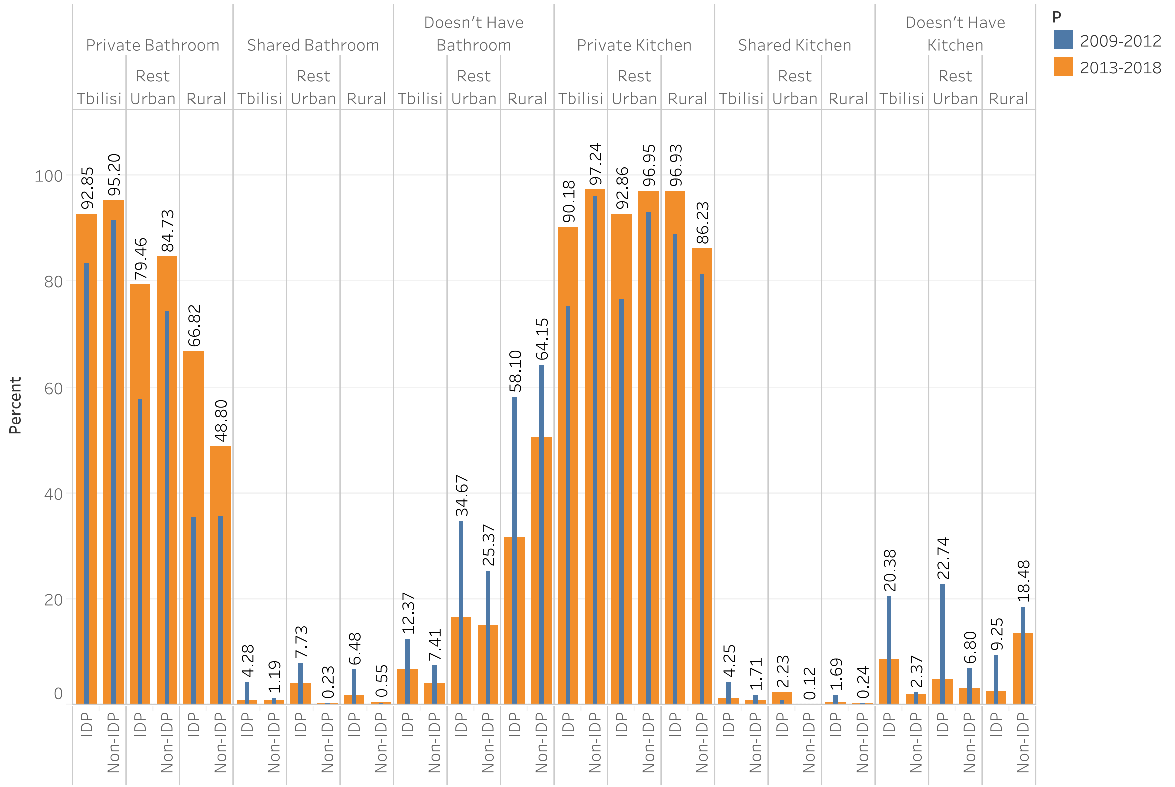

Figure 4: Access to kitchen and bathroom. Comparison of different forms of access to bathrooms and kitchens by IDP status, geographic area, and time period

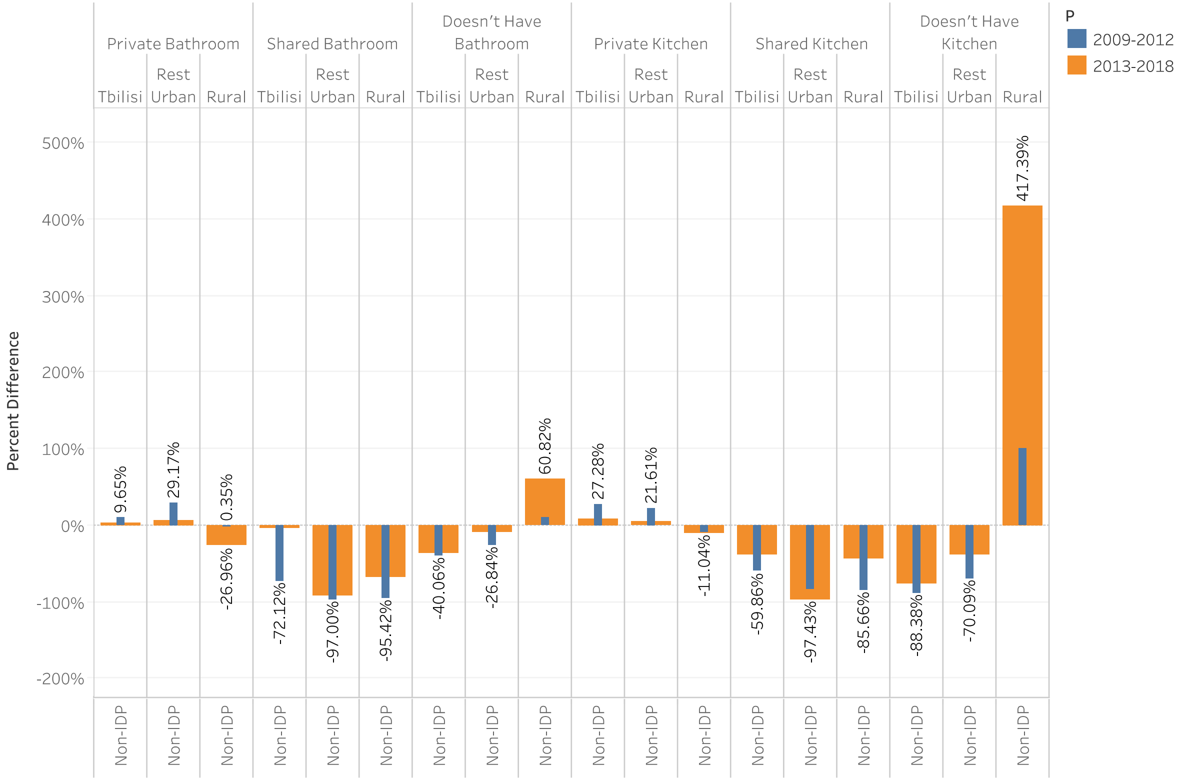

Figure 4: Percent difference of access to kitchen and bathroom

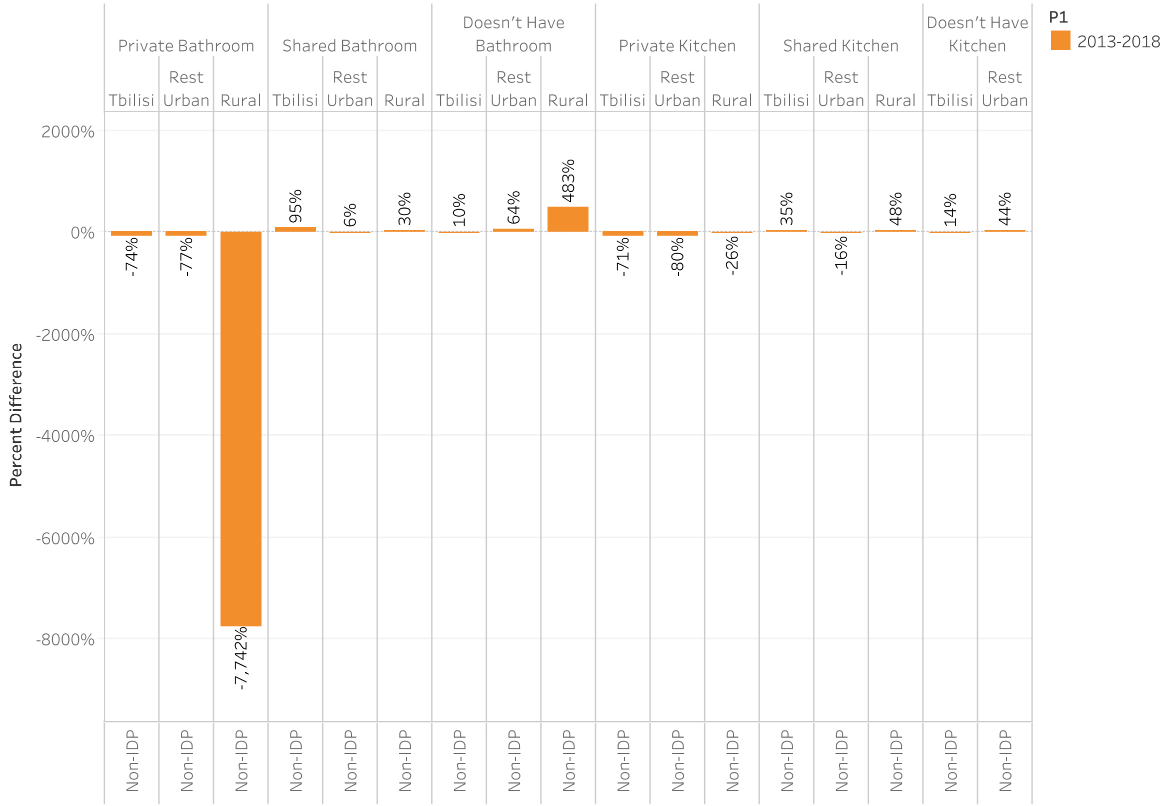

Figure 4: Change in percent difference of access to kitchen and bathrooms over time

Non-IDPs more frequently have a private bathroom and kitchen, and less often share or do not have a bathroom or kitchen compared to IDPs. There is, however, a major exception: non-IDPs in rural areas perform consistently worse than IDPs on nearly every measure. Rural non-IDPs less often own a private bathroom or kitchen and more often do not have either; this is consistent over time as well. Conversely, rural non-IDPs do appear to share bathrooms and kitchens less frequently. The gap appears to be closing in across all measures, with a major exception across rural non-IDPs, for whom the gap appears to be opening.

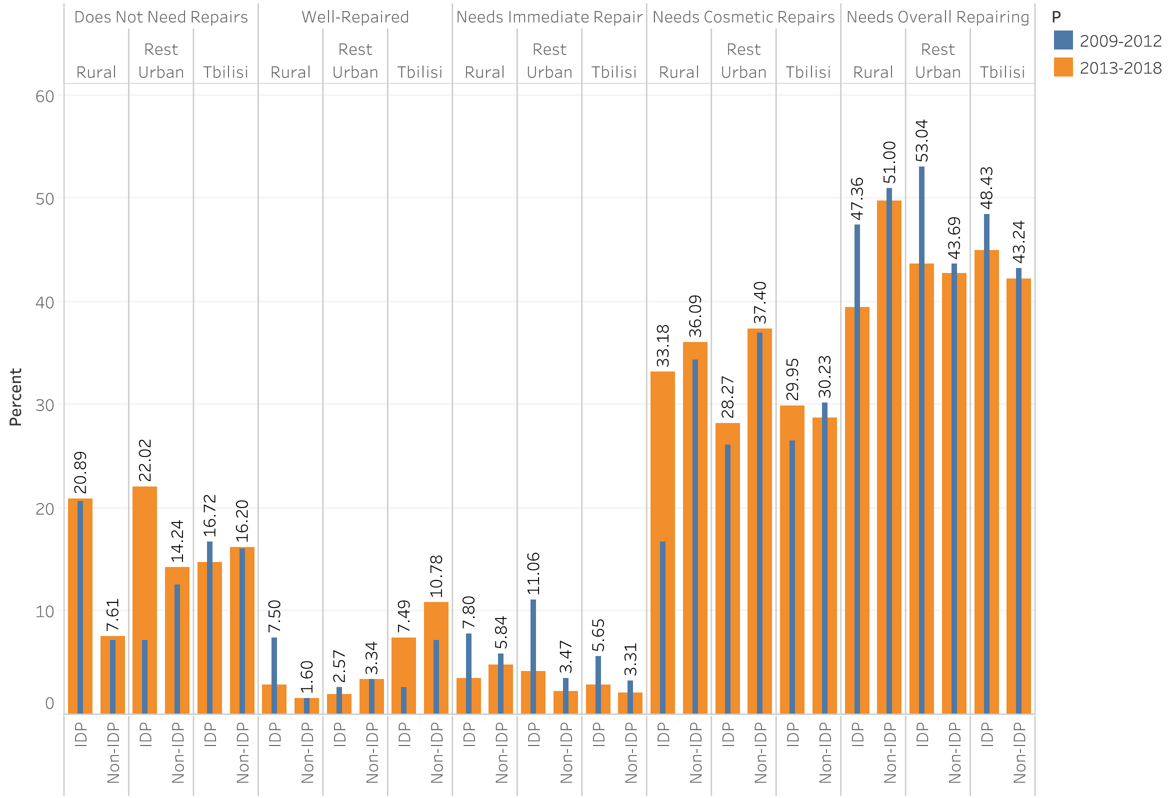

Figure 8: Status of home repairs

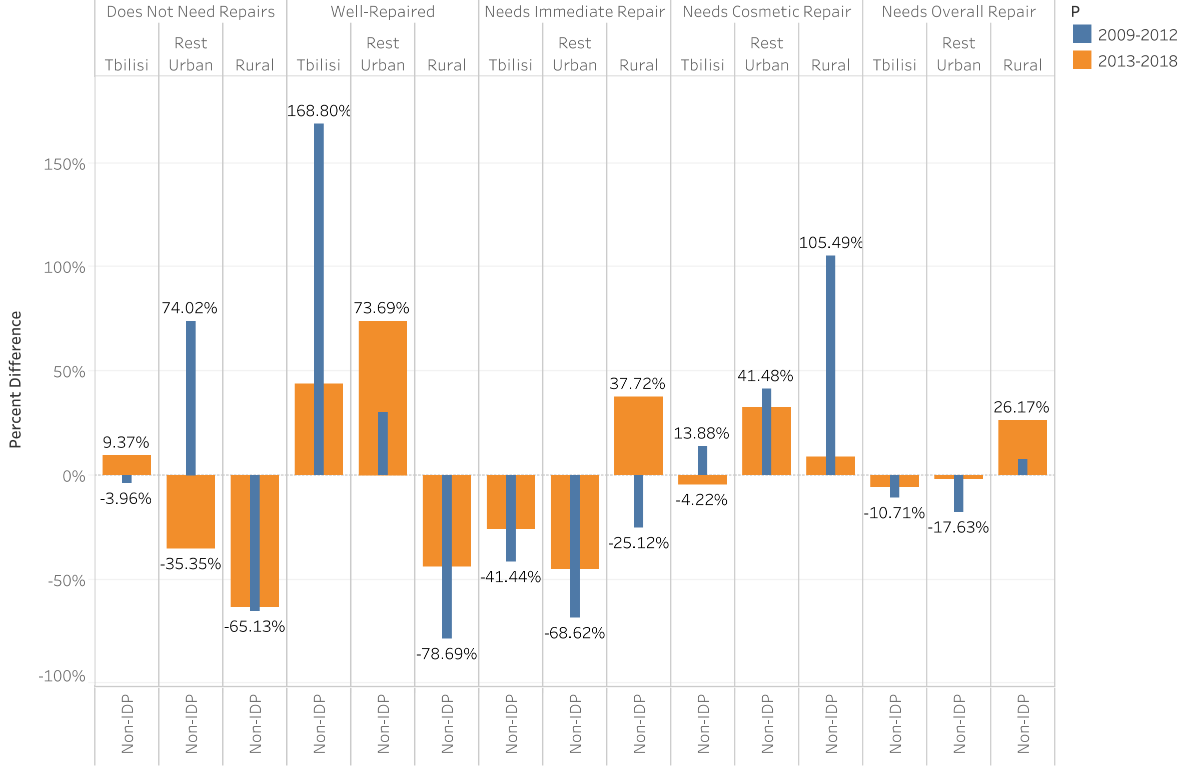

Figure 8: Percent difference in status of home repairs

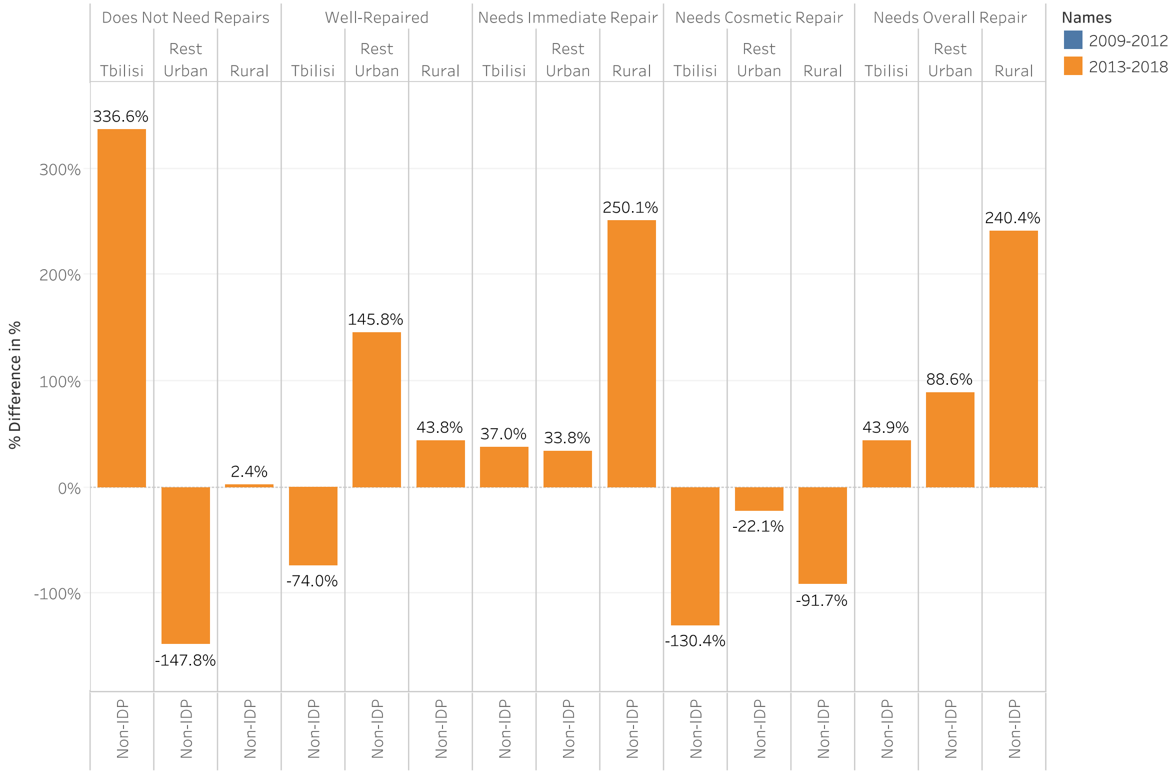

Figure 8: Change in percent difference in home repair status over time

A comparison between the status of home repairs between IDPs and non-IDPs is less obvious; there is greater variation between measures. Although, there are several important points to highlight. More non-IDPs perceive their repairs as well done 30%-168% more than IDPs, with a major exception in rural areas where IDPs are more satisfied by 40-78%. This trend largely carries over with the need to repair. Non-IDPs less frequently need immediate or overall repair on their homes compared to IDPs except in rural areas. Conversely, non-IDPs more consistently need cosmetic repairs compared to non-IDPs. Surprisingly, despite relatively consistent need for some repair, IDPs were more likely to not need repairs relative to non-IDPs, with some exceptions in geographic area and time period (Rest urban 09-12, Tbilisi 13-18). Whether these gaps are opening or closing also appears to be specific to geographic contexts.

Economic Instability

Income

According to unofficial statistics from 1998, 75% of displaced families earned less than half the monthly subsistence income level, set by the Georgian government at $35 per family member. [14] According UNHCR (2015), the average monthly income for a cohort of 2,001 IDPs

was 453 GEL per household, were 40% of respondents reported having an average household size of four to five persons. By contrast, the average monthly household income in Georgia is 887 GEL and the average individual income is 247 GEL – roughly double the income earned by IDPs. ISSA survey interviewers did, however, share their impressions that a considerable number of IDPs were hiding additional income other than official wages or pensions, which could have increased their reported income. Income levels vary across urban and more rural contexts, decreasing substantially from cities to villages and towns.[15]

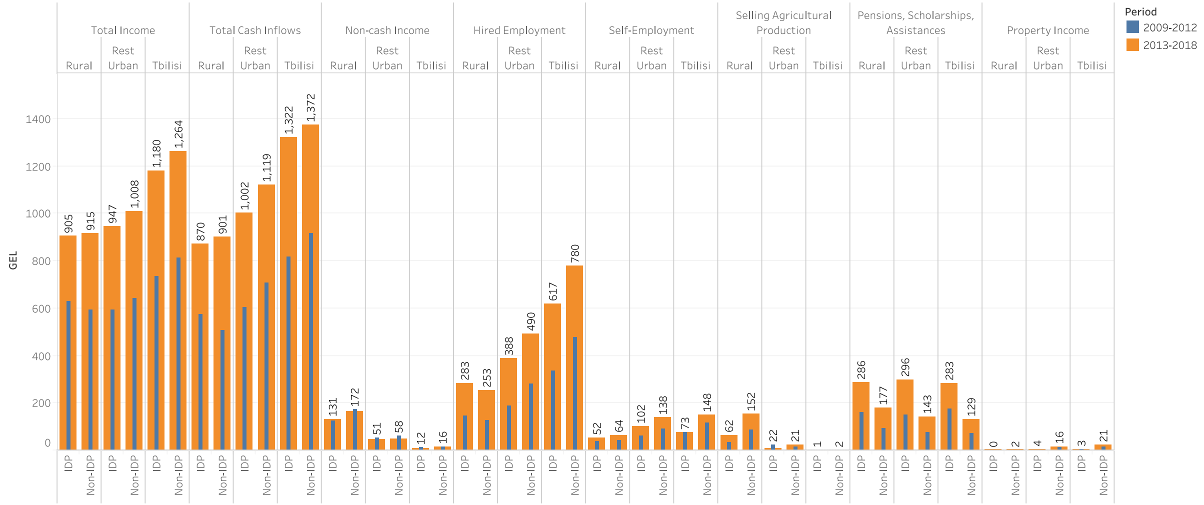

Figure 8: Income levels

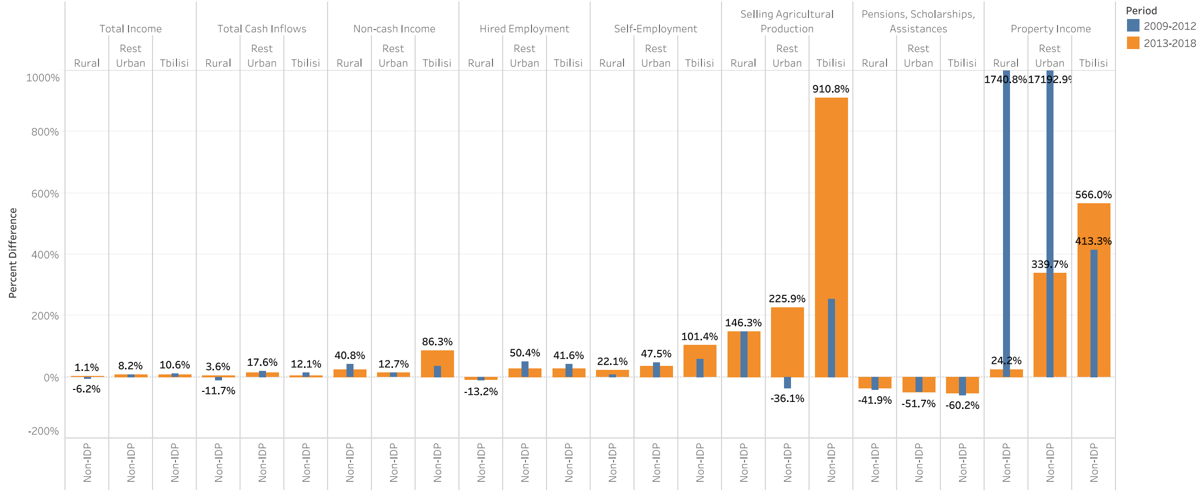

Figure 8: Percent difference of income levels

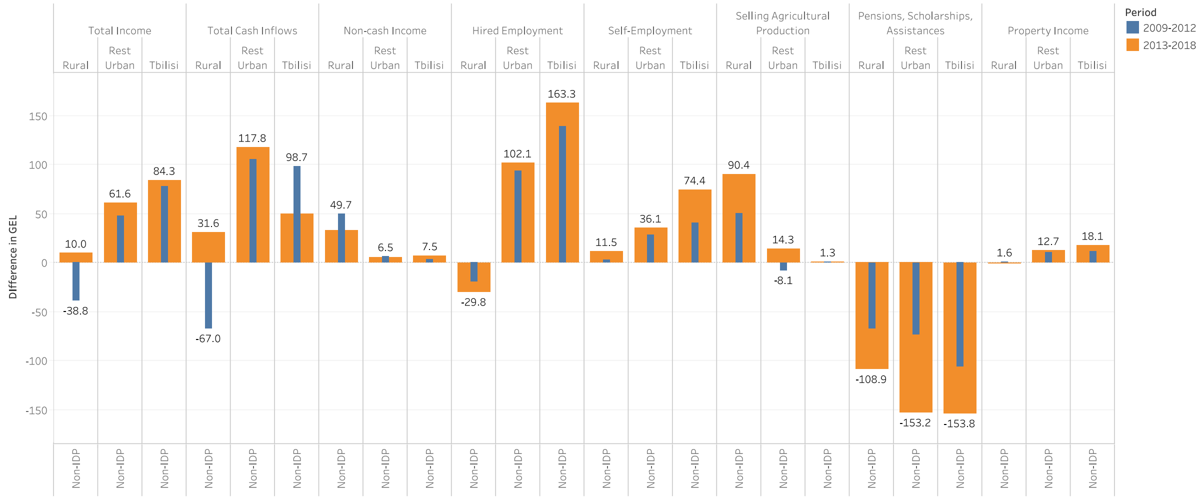

Figure 8: Difference of income levels in GEL

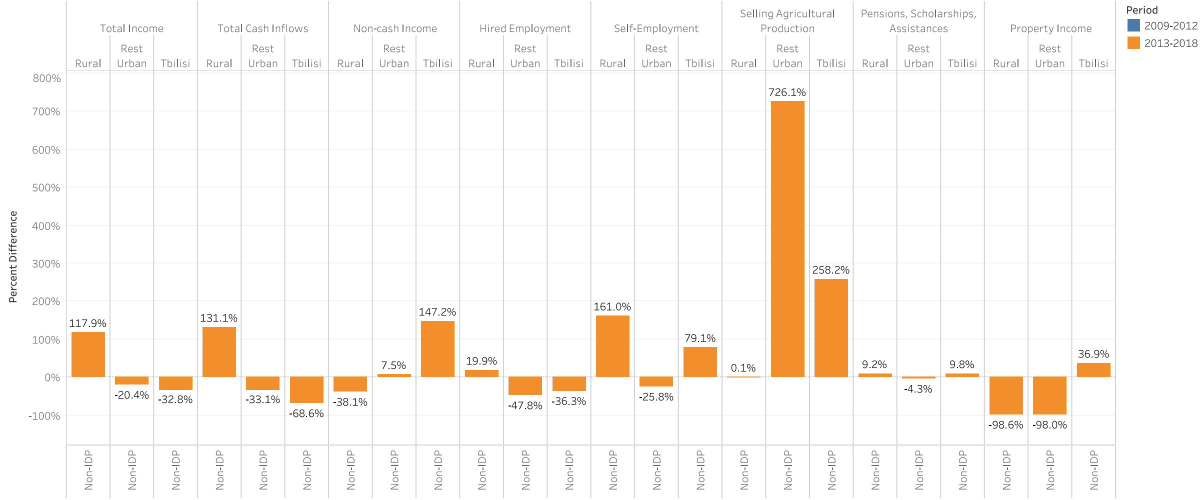

Figure 8: Change in the difference of income levels

Unemployment Rate

Unemployment is one of the primary causes of poverty among IDPs. Numerous reports have corroborated findings that IDPs, especially CC-IDPs, are acutely effected by unemployment compared to the general population. The UNHCR and NRC concluded in 1998 that upwards of 64% of IDPs were jobless. Of the female respondents to the CDIE survey, 68 percent were without work as of late 1999 and increasingly demoralized after nearly a decade of extensive joblessness.[14] SC (2002) found an unemployment rate of 40.2% among CC-IDPs in west Georgia compared to 23.1% for the general population.[3] Using similar criteria, the IFRC (1999) found an unemployment rate of 45% among CC-IDPs, compared to 31% and 15% for PA-IDPs and the general population, respectively; in other words, unemployment is 2-3 times higher among CC-IDPs.[4] Similarly, Tskitishvili et al (2005) found lower employment rates for urban IDPs compared to the GP surveyed between the early 1990s to 2005.[16] Nadereishvili and

Tsakadze (2008) found employment rates of 27.1% and 34.3% for CC-IDPs and PA-IDPs, respectively, compared to a 50.6% national average.[7] In the same year, DRC (2008) found 31.3% employment rate among 3,000 surveyed old caseload IDPs (roughly 1.3% of the old caseload population).[17] This is consistent with the 80% unemployment rate for old caseload IDPs found by Conciliation Resources (2011), which is 4.5 times higher than the national rate for that year (17.9%).[18] Interestingly, CARE International (2009) survey (500 respondents) found comparable rates for the newly displaced. Only 8% of those relocated to settlements in Kvemo Kartli and Shida Kartli identified themselves as being formally employed, and out of the 78% who identified themselves as not having any formal employment, 24% claimed that they had formal jobs before the war and 22% said they had marketable skills before the war – i.e former nurses, teachers and carpenters -- that were not used now. Asylum Seekers (2010) found that only 31% of the displaced working population was employed.[19] Over three-quarters (75.5%) of IDPs surveyed by CME (2013) were unemployed compared to the 15% national rate.[20] Further analysis of statistics provided by the Georgian Statistical Office (2013) suggests that urban IDPs are up to 3.5 times more likely to be unemployed than their non-IDP counterparts. It is also important to note that IDPs remain unemployed for longer periods of time compared to the general population. According to a 2013 World Bank study, the percentage of poor IDPs who have been unemployed for more than a year is double that of poor non-IDPs. Poor IDPs are also three times as likely to have never worked before, indicating higher levels of discouragement and limited integration in social and professional networks (IHS).[11]

The IHS reveals stark differences in employment between IDPs and the GP. Across all four measures of employment – economically active, employed, hired, and self-employed -- non-IDPs reported 15-112% higher rates in nearly every geographic area between 2009-16, with the exception of rural IDPs that had roughly 10% higher rate of hired employment in the same time period. Interestingly, across all measures and geographic areas this gap appears to be increasing overtime as high as 38-fold, except in other urban areas where the gap has closed by 17-98% under all measures except self-employment where the gap increased by 184%. Unemployment trends bear a similar resemblance. In every geographic period, non-IDPs were unemployed less (20-61%), for shorter periods of time (10-82%), and were more hopeful in their job search (48-75%) between 2009-16. Only two exceptions can be seen between 2009-12 where a greater percentage of non-IDPs: (1) lost hope searching for jobs in Tbilisi (41.25%), and (2) were unemployed for 1-3 years (13.59%) in other urban areas. Both numbers reversed in the subsequent time period (2013-16), although for different reasons. Whether these gaps are opening or closing overtime is less consistent, but points to several interesting observations. In Tbilisi, there is a drastic increase in the number of IDPs losing hope in their job search whereas this gap appears to be closing in all other parts of the areas of the country. Furthermore, the gap in unemployment and those who have never worked generally appears to be closing (25-44% and 1-24%, respectively). Changes in gaps with respect to length of unemployment is split fairly evenly, with 4 out of 9 measures showing the gap closing (3-54%) and the remaining 5 measures opening by 44-603%.

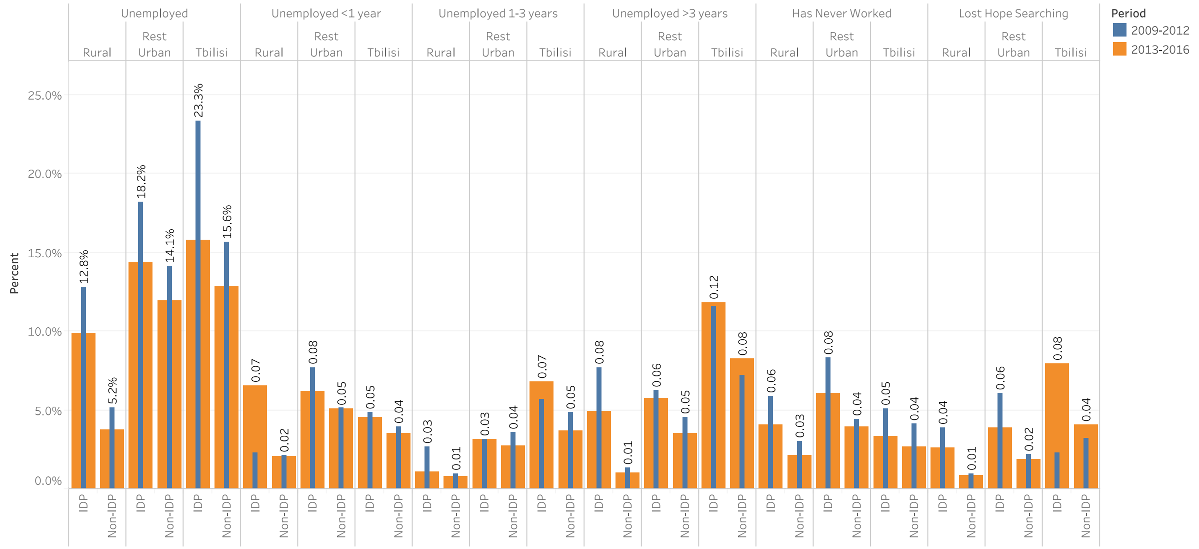

Figure 8: Unemployment levels

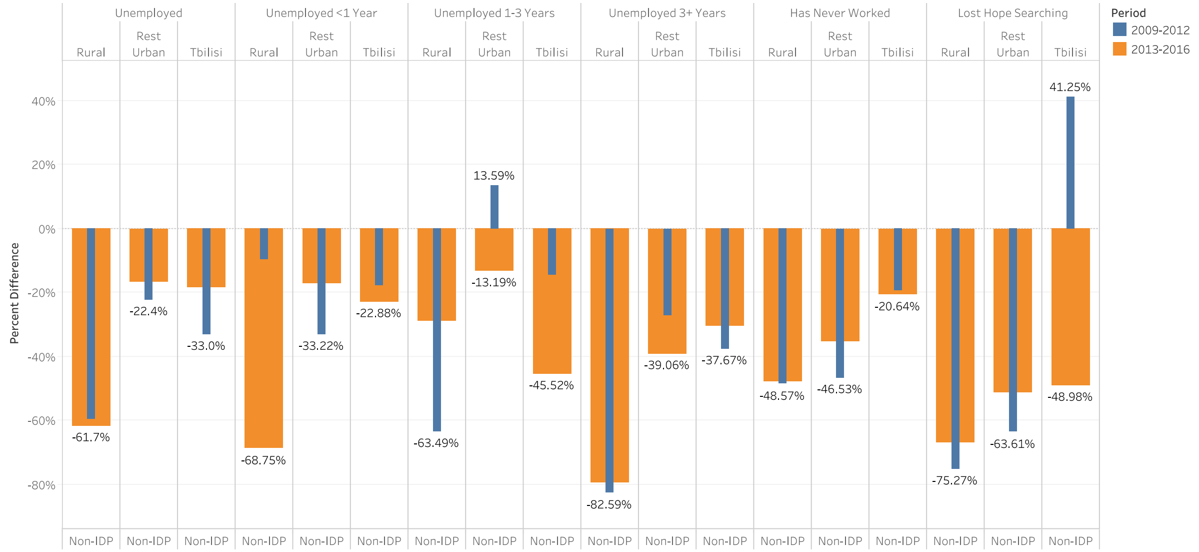

Figure 8: Percent difference in unemployment

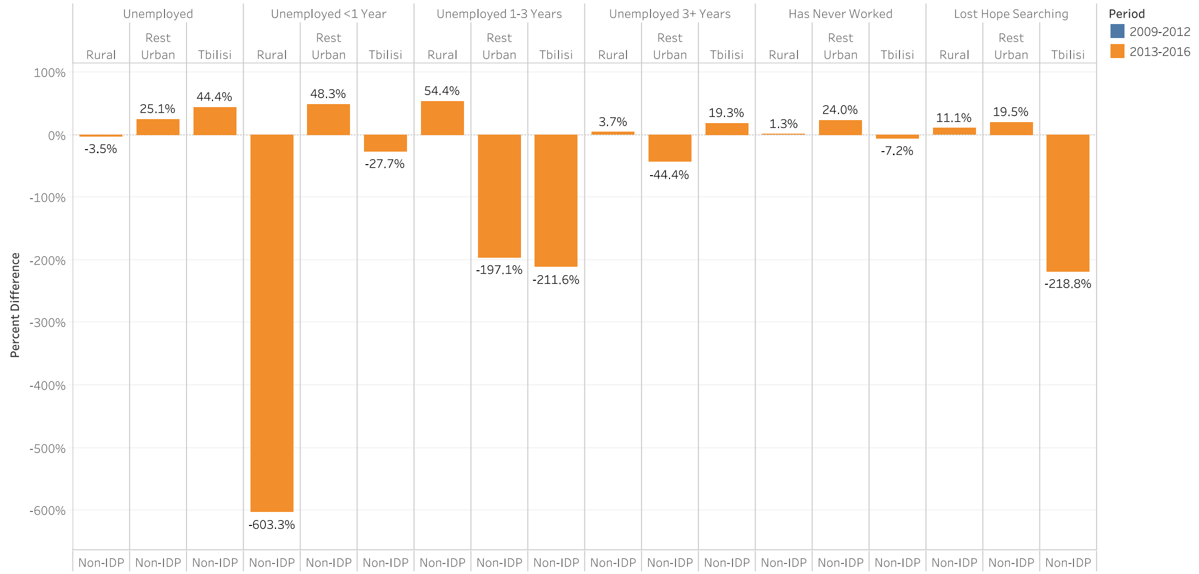

Figure 8: Change in difference of unemployment

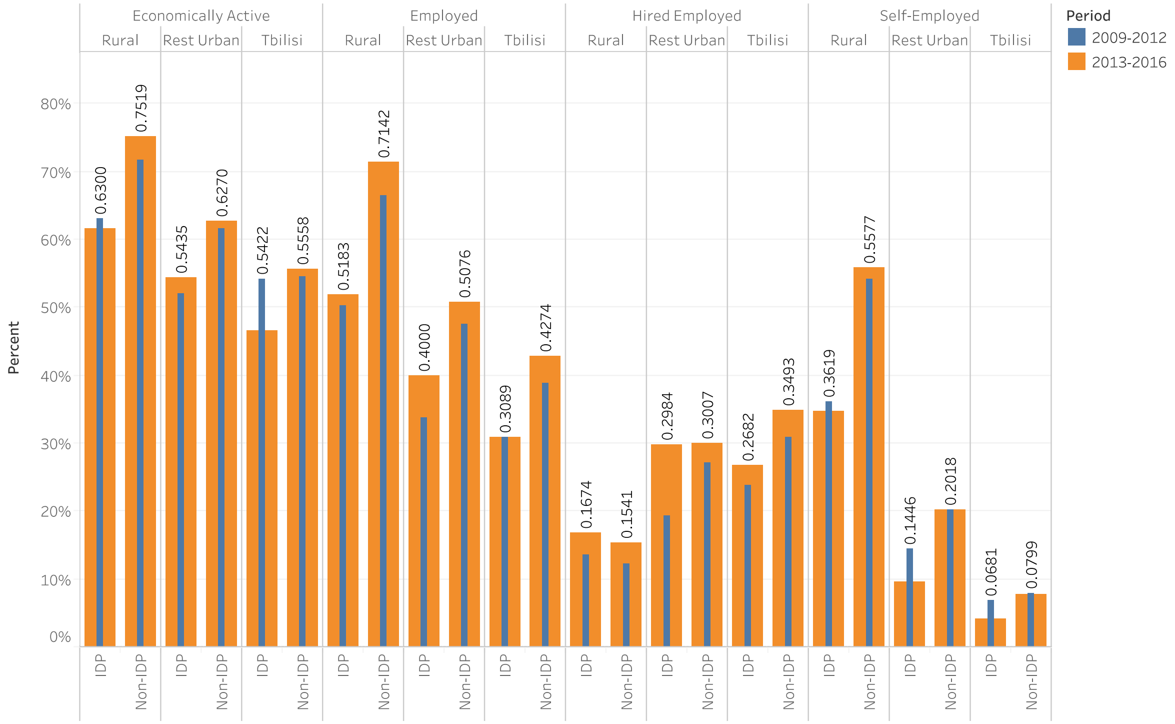

Figure 8: Employment

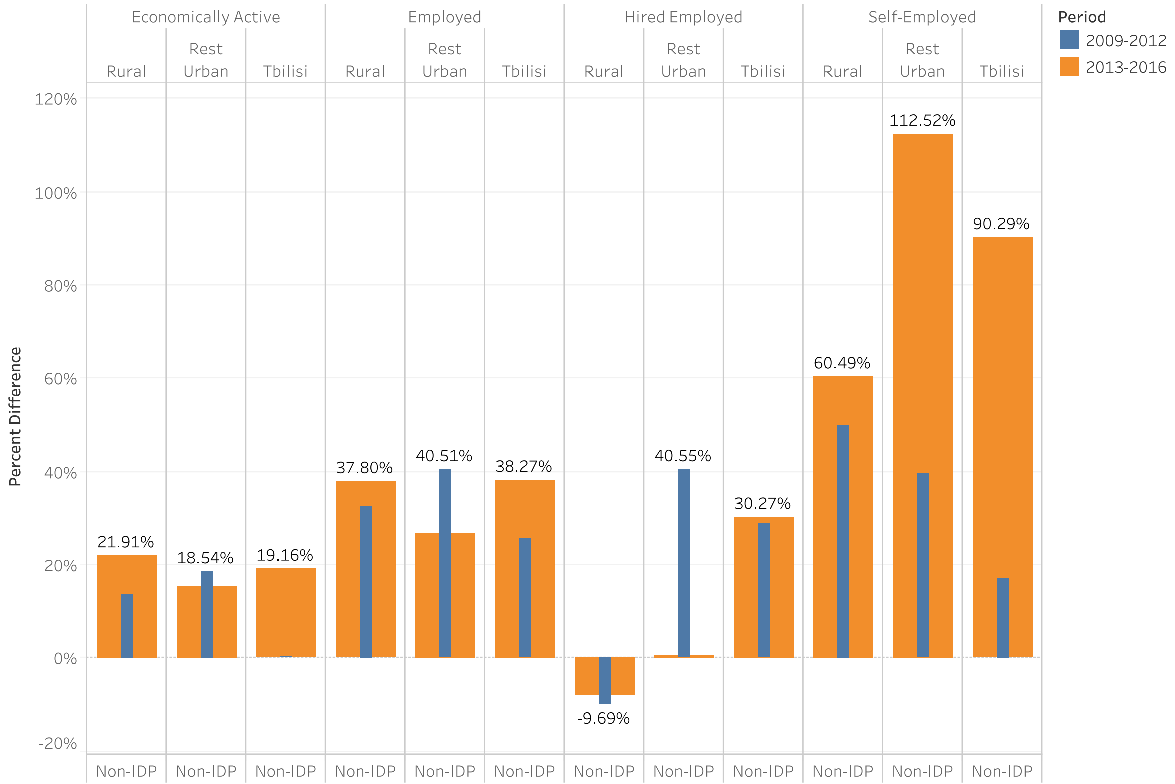

Figure 8: Percent difference of employment by status

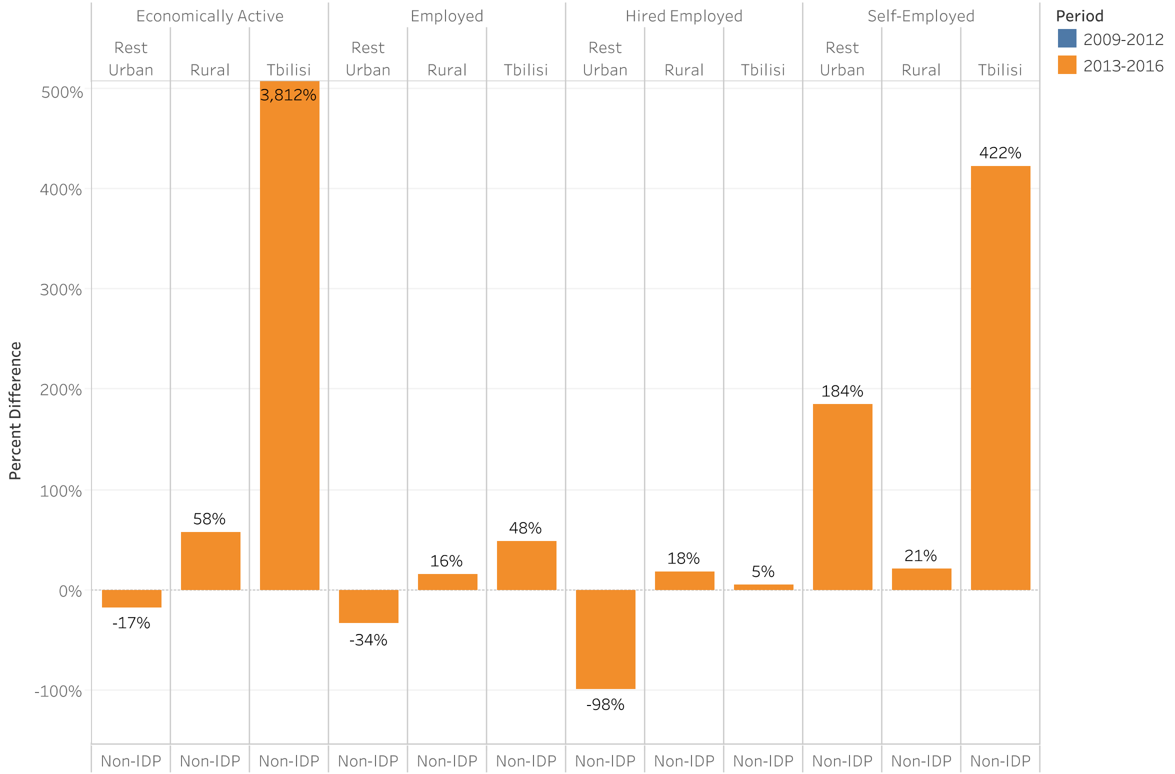

Figure 8: Change in difference of employment over time

Barriers to Employment

The remote locations of some CCs and new settlements combined with reported lack of infrastructure or access to usable land greatly reduces employment, self-employment, and other agricultural opportunities.[8 21 11] Large distances often separate CC-IDPs from land allocated to them or farmers' cottages, wholesale markets, and other various services and places of employment such as roads, transportation, pharmacies, schools, kindergartens, hospitals, banks, etc.[10 11] The spatial distribution of CCs also poses a major barrier to social integration, which has an acute impact on employment. This leads CC-IDPs to depend more on localized social networks, which can inhibit them from gaining information about skill-enhancing or employment opportunities and demotivate them from actively looking for a job when opportunities are scarce in their settlement. Although some social capital is built through contacts with non-IDP communities (particularly through children attending schools), often times they can be superficial and rarely result in employment opportunities.[12 21] Gogishvili notes that displacement swelled the local population size of major cities by 20-30%, which put pressure on the job market and infrastructure.[21] Moreover, the lack of long-term housing security means these can be severed again if IDPs are resettled out of the area. It is important to note, a majority of IDPs report preferring to be relocated to new living spaces, but largely prefer not to migrate from the region village or city due to concerns over socialization and integration.[15] Thus, migration in pursuit of jobs is limited and particularly lower among non-resettled CC-IDPs relative to IDPs living in private accommodations, although Migration Research Center (2012) characterizes displaced families by a high intensity of labor migration.[22] The DRC (2008) also reports particularly long duration of labor migration (nearly 8 years on average).[23]

Many IDPs do not have relevant skills need to adjust to the labor market, including proficiency in English and computer-based skills. The protracted nature of displacement, which involves extended periods of time without education, apprenticeships, work experience and training, leaves many IDPs uncompetitive in the job market. This is especially problematic for those displaced at a younger age prior to gaining any foundational education or work experience [11 24]. Even so, skills that may have served IDPs in their home region may not be relevant or applicable in their new environments, and typically the depreciation of skills not only decreases the labor force in numbers, but also leads to an emergence of a less-skilled group of people with a loss of self-confidence that further perpetuates their social exclusion from the labor force[12]. IDMC (2009) reports some IDPs accepting jobs that they are overqualified for or demand different skills.[25] Some NGOs also report that IDPs have unrealistic expectations of the types of jobs they are qualified to do, which negatively impacts the jobs they seek. Interestingly, some experts (e.g. at ACH) observe that IDPs still frequently refuse to participate in vocational and business trainings in part due to skepticism over their utility. Mistrust can sometimes be seen in IDPs who have already participated in vocational training and refuse to participate in another when it is not paired with a job or assistance in finding a job, which otherwise renders the training useless and not worth the effort. In some cases, IDPs who have undergone vocational training, would not receive certificates or diplomas that could facilitate finding a job.[11]

Interestingly, the resettled population appears to have been a predominantly agricultural prior to their displacement. A study that covered 22 new settlements in Shida Kartli and Kvemo

Kartli, Georgian regions bordering the conflict zone, South Ossetia, revealed that 76 per cent of the interviewed were engaged in agriculture before the war, while this number had now decreased to 40 per cent, due to clear reasons including insufficient and low quality land and a lack of access to irrigable water.[12] Limited access to land prevents CC-IDPs from cultivating products for sale or for their own consumption. According to SC (2002), IDPs living in collective centers are 3½ times less likely to have access to land than the local population, and 2 times less than IDPs living in private accommodations. And, for the few IDPs that report having access to land, it is generally quite small; approximately 400m2 on average reported by IFRC, which is 3 times less than the amount of land used by IDPs living in private accommodations and 6 times less than the local residents.[3] A 2007 government ruling that local authorities may allocate state agricultural land to IDPs, although the process of selecting plots and distributing them is left to the discretion of authorities and has not been consistently applied. Importantly, if IDPs stop using land plots given to them they are transferred back to the local administration[7 6]. Land rental is a common practice in rural areas, but some IDPs are reluctant to utilize this opportunity due to lack of trust in landlords and a lack of knowledge and understanding surrounding rental agreements.[11] Reportedly, many are unable to participate at all in the special auctions held for the sale of agricultural land, either because they lack funds, or because they are only registered with a temporary address and not in the community registries[8]. Cultivating land also requires secondary resources and equipment that are also difficult for IDPs to obtain. Limited funds also prevent them from buying agricultural equipment and livestock. New caseload IDP settlements have a noticeable lack of agricultural storage facilities, forcing crops to be sold in bulk and cheaply before they rot[11]. Lastly, even in cases where farmers were provided with arable land, studies report that only a few households are able to support themselves exclusively with their farm. This highlights additional business-related problems. For instance, the presence of overly competitive markets and a lack of technical understanding about constraints or opportunities for marketing, profitability, and how to adapt activities to achieve more income. Strained trading relations with Russia also adds significant barriers to IDPs trying to access markets to sell goods[11]. Altogether, a combination of poor infrastructure, inadequate storage facilities, and a lack of access to markets and processors make it hard for IDPs to engage in profitable agricultural enterprise.

Access to financial assets, such as micro credit, is an important prerequisite to the accumulation of other assets. While it is true that people were experiencing difficulties in obtaining credit even before the conflict erupted, conflict further deteriorated the situation, since lending institutions simply ceased giving out loans altogether or refused to provide credit to those farmers who were unable to repay their credit from previous years as a result of the conflict. Only 8 per cent of the 470 IDPs interviewed applied for a bank loan and only 10 per cent of the loan requests were made for business development. The rate of the bank applications was double, though also low, for the non-IDP host population. Focus group interviews revealed people hesitated in taking loans for starting a business citing the lack of money and the lack of market demand as the main obstacles. The main reasons of refusal for a loan among those who applied and did not get the loan were low income and too little collateral, as well as a lack of business skills or experience.[12 19] This highlights the importance of home ownership as a point of entry for entrepreneurial IDPs trying to take advantage of economic re-entry initiatives; for instance, a one billion GEL rural and agricultural subsidized credit scheme advanced by the government aims at providing interest-free business development loans and cost-sharing grants open to IDPs, but requires collateral to participate[11]. A lack of trust in financial institutions,

misperception of unfair credit distribution, high interest rates, and the fear of losing collateral constitute additional reasons for why taking bank loans might appear to be a risky endeavour to IDPs.[11] [12] Reports have recommended taking a more tailored approach to microfinance by providing collateral-free credit and financial education based on local and individual needs. There are several organizations allocating grants or loans – for instance, microcredit funds that offer subsidized credit at interest rates reduced by upwards of 50% -- for income generating activities primarily to existing businesses, although some support businesses operated by first-time or unexperienced owners. Some NGOs have also partnered with enterprises to provide business development training to accompany financing schemes that involve grants or credit.[11]

Psychological dependence on social benefits can also negatively impact financial independence. The prospect of benefit reductions can produce fear that compromises their desire to find employment. A lack of financial independence is especially impactful on the self-esteem of IDP men who generally find it more difficult to secure employment; in part because of an unwillingness to do menial work or participate in vocational training that is not as pronounced in women. Aside from the cultural stigma associated with financial dependence, a poor understanding of the social allowance system and eligibility criteria might also contribute to misconceptions that prevents IDPs from taking jobs, particularly in the formal economy. The psychological strain further imposed by financial worries and an uncertain future might also impede positive practices for job searching and employment.[6]

Poverty:

Almost one half (48.1%) of IDP households also stated that their income (or their harvest) is enough only for nutrition, while only 17.3% of households are more or less satisfied with their economic condition.[6] CME (2013) reports that 66% of their respondents identified themselves as "poor" or "very poor" based on their income[20], Nadereishvili and Tsakadze (2008) report that every sixth IDP household regards itself as extremely poor and claimed to be constantly starving, while approximately the same number of households claim they hardly manage to feed themselves.[17]

Many studies also report that IDPs have substantially fewer productive assets and durable goods than the general population. CC-IDPs appear to possess fewer durable items – i.e. TV sets, refrigerators, mobile phones, clothing, etc. – and are 2-6 times less likely to own various livestock (poultry, pigs, and cows) and equipment (cars, tractors, and sprayers)[1] compared to the local population.[16] [17] World Bank (2017) also reports that PA-IDPs are much better-off in per capita consumption than CC-IDPs[26].

Disparities in access to infrastructure are also noteworthy. There are concerns over access to adequate heating which may either be unavailable (firewood and gas) or too expensive (electricity). The government provides assistance for communal expenses such as electricity, water consumption and disposal, but can sometimes be insufficient if it is also used for heating. Thus, with insufficient income, some services may be covered at the expense of others; for

[1] SC (2002)

instance, waste disposal (especially in rural areas) maybe irregular and lead to hazardous unsanitary conditions. Moreover, CCs typically have a common electricity meter or no meter at all, which leads to frequent abuse and can provoke electric companies to terminate their services when the bills are greater than the government subsidy[7].

The multidimensional poverty index (MPI) incorporates four non-consumption-based dimensions: demographics, education levels, employment status, and access to infrastructure. According to this measure, the national general population sample is substantially better-off than IDPs. Interestingly, the share of respondents who may be considered ‘multidimensionally poor’ according to the MPI is similar between old and new caseload IDPs (23% and 21%, respectively).[26]

IDPs are also worse than the national sample in other non-consumption-based measures; IDPs live in smaller dwellings, have less adequate housing, own their home less, and are more reliant on social assistance. IDPs in new settlements also generally have less social capital than their host population, and existing social cohesion can be disrupted by the possibilities of tension over access to land, water, firewood, infrastructure, and jobs.

IDPs are the beneficiaries of several targeted assistance policies. Prior to legislative reforms in 2014, PA-IDPs received an allowance of 28 GEL per person per month; those in CCs receive 22 GEL. Since then, the allowance has increased to 45 GEL and is given to both CC-IDPs and PA-IDPs.This is in addition to a one-off cash assistance for newly displaced persons and entitlement to free health insurance so long as qualification criteria related to the level of poverty are met. Moreover, IDP families living below the poverty line are eligible to apply for the Targeted Social Assistance (TSA) program, launched by the Government in 2006 to alleviate poverty among extremely vulnerable segments of the population. If they opt to, IDPs can be assessed for TSA eligibility, through which the head of household receives 30 GEL/month and remaining family members receive 24 GEL/month; although, IDPs claiming TSA are required to give up their IDP allowance. As of February 2019, 84% of IDPs receive the state IDP allowance of 45 GEL, and 11% receive TSA. Overtime, CC-IDPs’ have become *more* dependent on these welfare programs. CME (2013) reports only one-third of survey respondents named a salary from employment as one of their main sources of household income, with many instead relying on state pensions (mentioned by 40% of respondents), or monetary assistance for IDPs (68% of respondents). IDPs in general report sources of income primarily derived from forms of welfare assistance: IDP-specific allowance (79.7%), old age pension (42.0%), earned wages (41.5%), social allowance (19.8%), war veteran pension (4.8%), among others. Reliance on state-sponsored assistance is also greater than the national average (45% of IDP families compared to 33%). 12% of IDPs receive TSA compared to 9% of the GP.

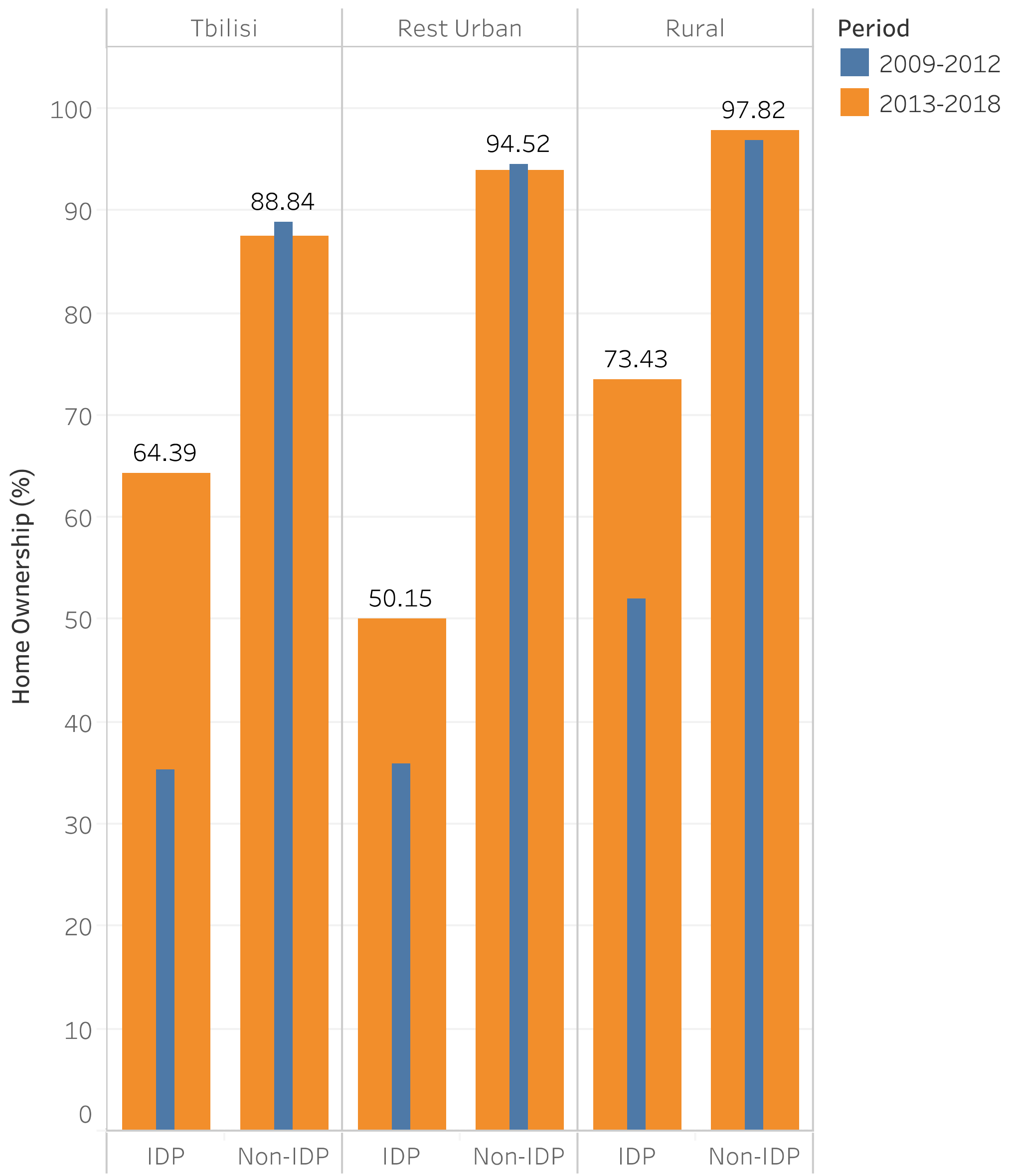

Figure 8: Home ownership

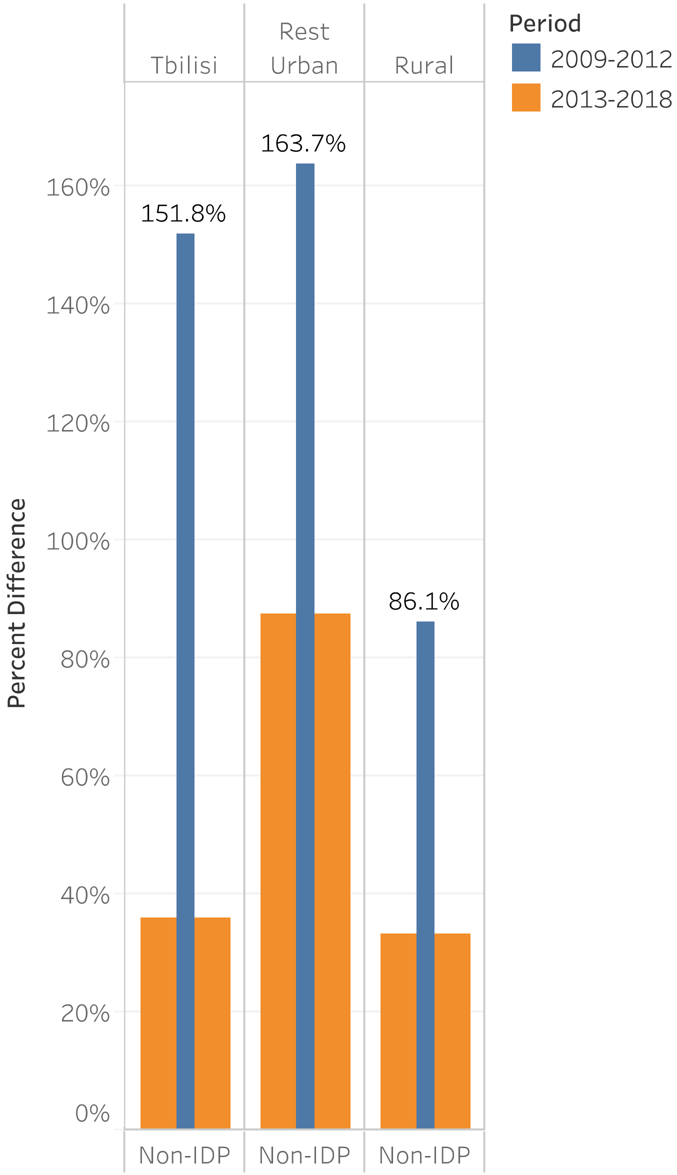

Figure 8: Percent difference in home ownership

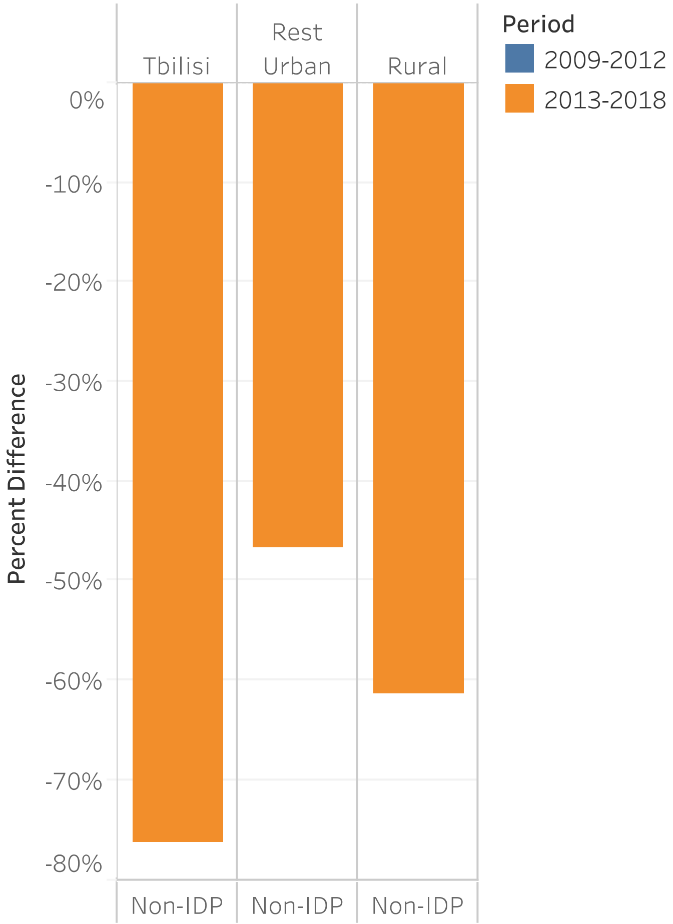

Figure 8: Change in difference of home ownership

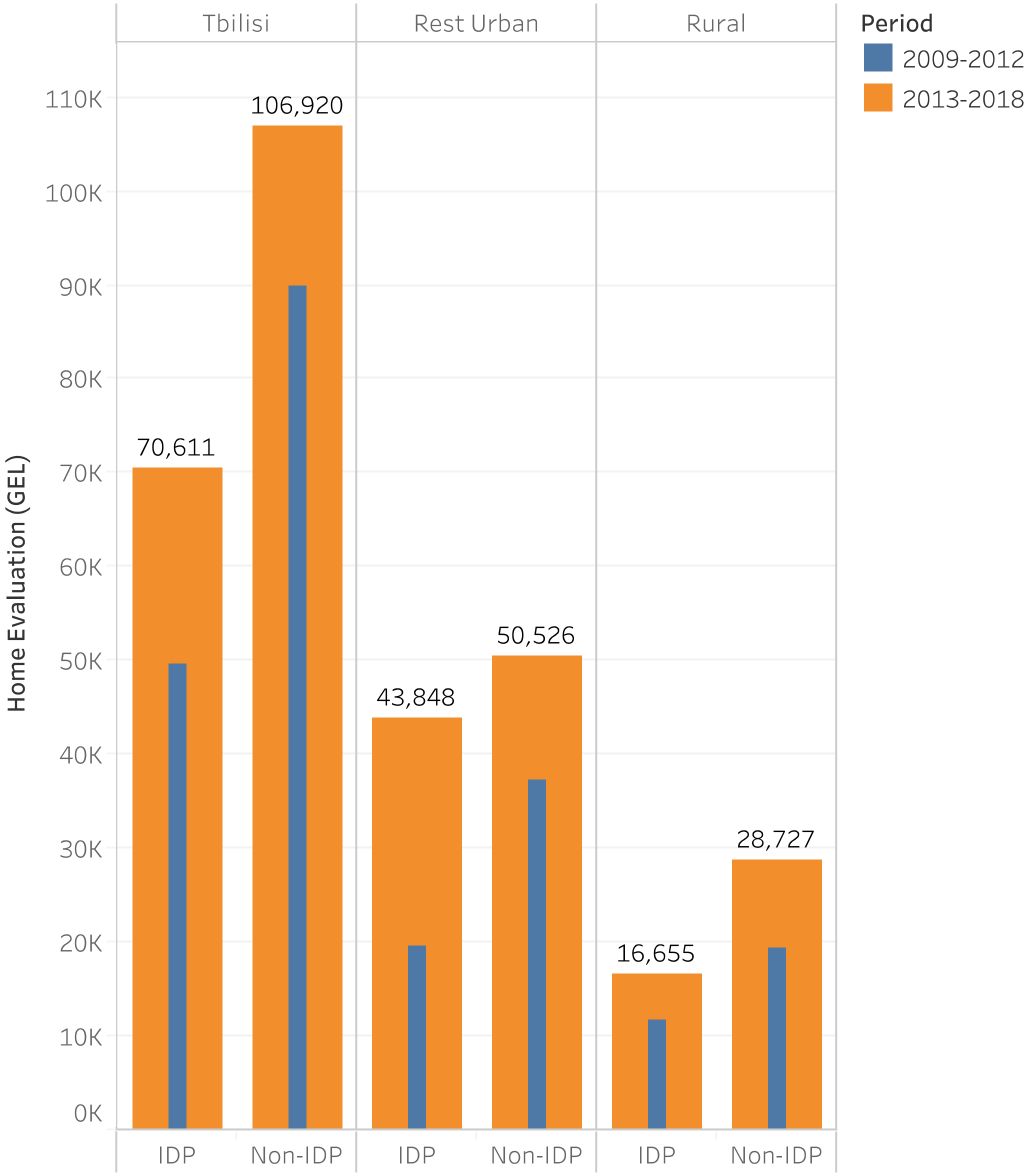

Figure 8: Value of homes

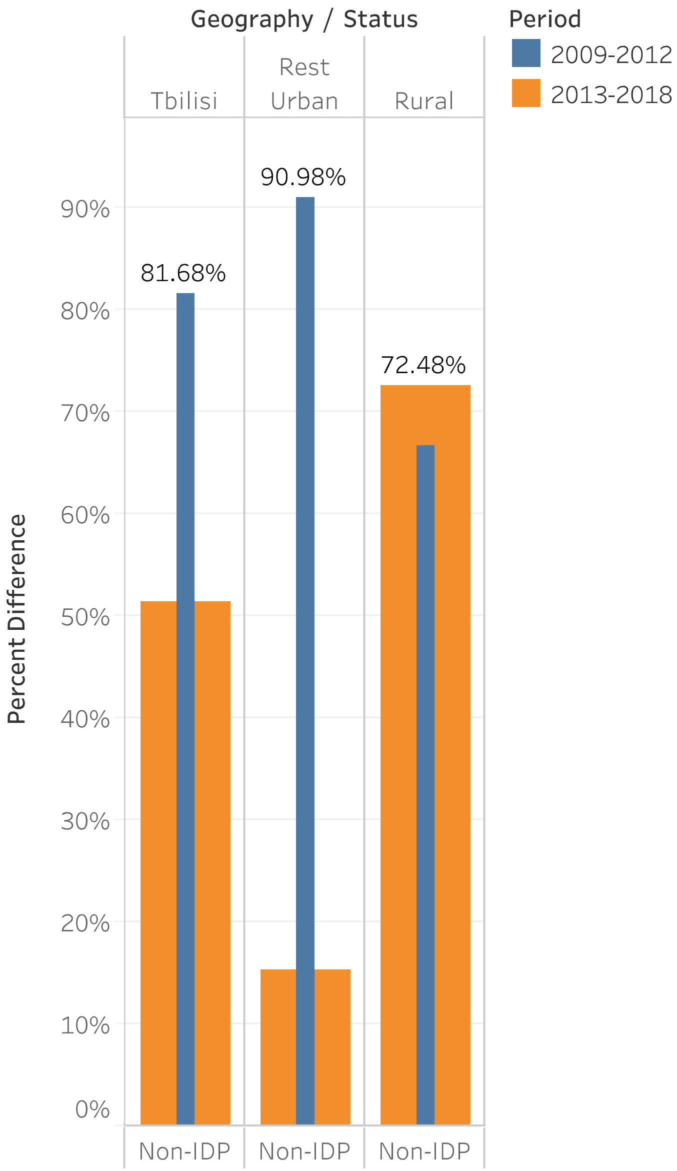

Figure 8: Percent difference in home value

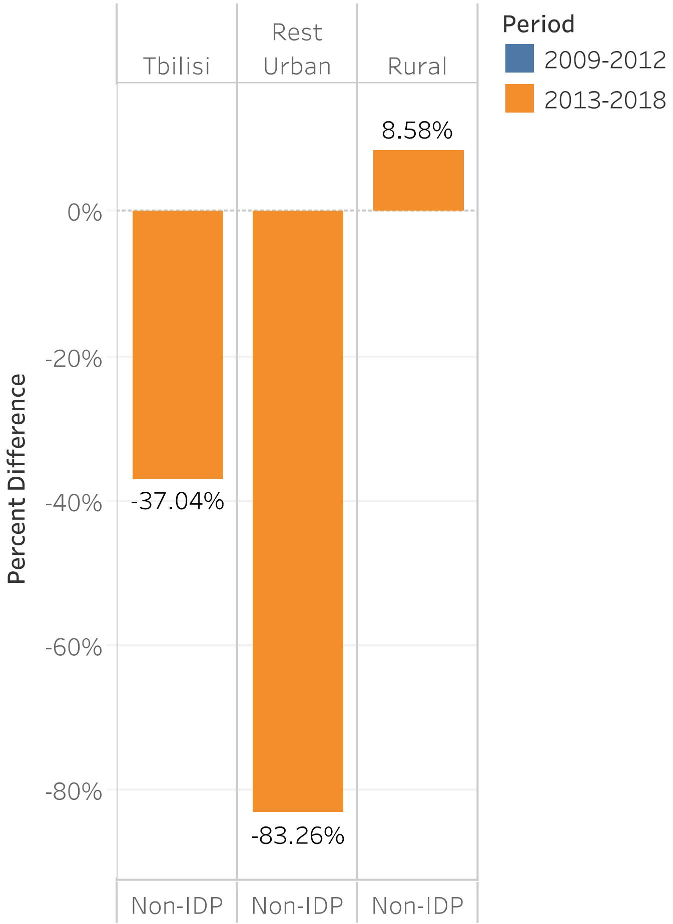

Figure 8: Change in difference of home value over time

The IHS (2009-2018) also reveals positive trends in home ownership, dwelling size, and value. Across all geographic areas, IDP home ownership and value increased by 40-82% and 42-124%, with the largest improvements seen in Tbilisi and rural areas, respectively. Ownership is not only extremely valued culturally, but an important asset for accessing financial institutions and securing tenure in an underdeveloped and volatile rental market. The total dwelling size has remained relatively unchanged for IDPs in rural areas, with more substantial improvements in Tbilisi (25.81%) and other urban areas (8.82%). In all measures, non-IDPs fared significantly better than IDPs across both time periods, which is consistent with other reports. [2] However, it is interesting to note that while a clear inequality gap still exists, in many instances the data indicates that it is closing. The difference in home valuations decreased by 30% and 76% in Tbilisi and other urban areas, respectively. Across all geographic areas, the difference in home ownership decreased by 53-115%, and in Tbilisi, the difference in dwelling size decreased by 28%. It is concerning to see a reverse trend in rural areas with respect to dwelling size (+9.15%)

[2] National surveys reveal that IDPs in general live in significantly smaller dwellings (59m^2 compared to 104 m^2 for the national sample), have less adequate housing (58% to 61%), and own their home less often (66% compared to 90% of the time). – G http://www.parliament.ge/uploads/other/77/77112.pdf

and home valuations (+5.72%), pointing towards a slower rate of improvement relative to non-IDPs in the same area.

History of Substance Use

In 2006, a damage reduction network was established which, by 2017, has since expanded to include 10 NGOs operating 14 service centers in 11 cities: Tbilisi, Gori, Telavi, Kutaisi, Samtredia, Poti, Zugdidi, Ozurgeti, Batumi, Sokhumi, and Rustavi. Mobile outpatient assistance expands the geographic coverage of the network to an additional 44 cities and nearby villages. The damage reduction network provides a suite of services, including: sterile needles, condoms, informational material, pre- and post-counseling and screening for HIV/AIDS, Hepatitis B and C, and syphilis, access to various medical specialists (therapists, psychologists, gynecologists, urologists, general practicioners, etc.) and lawyers, and counseling for sexual partners or individuals on healthy sex practices and relationships. It is critical to note, however, that the availability of services differs across cities. A recent multi-city survey revealed that coverage of preventive programs -- defined as knowing where to get an HIV test and receiving at least one of the following program commodities: sterile injecting equipment, condoms, and qualified information on HIV/AIDS through brochures, leaflets, and booklets -- had increased from 24% to 32.4% since 2012. The composition of preventive packages also differed across cities, with the most extensive program coverage found in Gori. For instance, sterile needles were received by approximately a third of Gori respondents (30.1%). Full-service coverage of the target population ranged from a low of 8% in Telavi to a high of 26.4% Gori. In general, coverage with preventive programs in the cities and towns studied was lower than the recommended minimum coverage. Importantly, it is unclear whether harm reduction services, treatment resources, and preventative health education are accessible in collective centers.

Mental Health

A consistent and dramatic mental health gap can be seen between genders. with women exhibiting higher rates of all conditions: 25.17% v 19.31% (PTSD), 14.12% v 10.26% (depression), and 11.35% v 8.49% (anxiety) (Comellas 2015). This was corroborated in Saxon (2017) – 26.4% v 19.2% (PTSD), 16.0% v 11.4% (depression), and 12.7% v 7.7% (anxiety) – as well as JHU (2012) in an exclusively older (65+) sample – 75.2% v 57.7% (depression) and 78.4% v 59.5% (GAD) and SC (2002). Similar gender differences can also be seen in terms of the severity of somatic distress, with women exhibiting higher rates of high (4.12% v 1.92%), medium (17.55% v 8.81%), and low (33.97% v 24.91%) SD severity; although, men demonstrated significantly higher minimal SD severity (64.30% v 44.34%) (Comellas 2015). Although not comparative, a 1995 Oxfam study rigorously examining displaced women and children in CCs concluded that over 86% of adults suffered from PTSD and its various symptoms, including heart and cardiovascular diseases (21%), chronic migraines and long-lasting sleep disorders (51%), and severe depression (22%). Similarly, 21% of displaced women surveyed by USAID in 2000 had been clinically diagnosed with a form of neurosis (Kharashvili 1995, 24–29; Zurikashvili 2000, 7). There is, however, another important distinction to be made

in how the trauma of displacement has effected men and women differently. UNHCR (2008), Buck (2000), and FDHR (1997) find that women have been much more successful at adapting to everyday life. FDHR (1997) noted that men were more fixed on returning to their past lives, and were characterized by patterns of escapism through growing alcoholism and empty and routine activities to pass time. Spouses indicated that their husbands and other men were doubly traumatized by the conflict, taking responsibility for losing the war and allowing abandonment, exile, and destitution on their families on the hand, while failing to fulfill their traditional role as leaders and breadwinners of the family; many reported having profound shame that women had become more creative in securing sources of income through various ventures.

Differences in IDP caseload are less clear, but still notable. Makhashvili reports higher levels of PTSD, depression, anxiety, and comorbidity of these conditions among OCL IDPs relative to NCL (27.1% v 22.9%, 18.7% v 9.9%, 13.0% v 9.2%, 16.0% v 9.8%, respectively. However, the opposite trend was observed by JHU (2012) among older IDPs (65+), with NCL exhibiting higher rates of GAD (76.6% v 70.3%) and depression (71.9% v 69.4%); although, the latter difference was not significant. The study population in JHU (2012) was also exclusively IDPs over the age of 65, as opposed to varied age groups in Makhasvili (2014). This might suggest that the difference between caseloads minimizes with older age, which, in turn, indicates that the effect of displacement on the elderly is acute regardless of the length of displacement. The difference between caseloads may be accounted for by greater hardship of displacement in the 1990s indicated in, for instance, higher exposure to traumatic events (i.e witnessing murder) and poorer living conditions (smaller dwellings, less adequate housing, etc.) [19]. It is important to note the strikingly (nearly four times) higher rates of symptoms in older IDPs (JHU 2012). Makhashvili, Comellas, Chikovani, and SC (2002), while not reporting prevalence in older age groups, notes that the risk of PTSD, depression, anxiety, and somatic distress increased 1.5-5.5 fold with age. This might be accounted for by factors that are conflict and post-conflict related. Mental disorders may become entrenched over a sustained period of time with lack of access to adequate care and treatment (Makhashvili and van Voren 2013). Ongoing impoverishment and poor living conditions may also exacerbate existing conditions. Insecurity of tenure, loss of home, job, and identity, the uncertainty associated with the collapse of the Soviet system (for older IDPs), lack of money, and a lack of optimism over the future impose enormous psychological strain on adults (Tarkhan-Mouravi 2009, Makhashvili, Tsiskarishvili et al. 2010, Miller and Rasmussen 2010). Interestingly, a study by FDHR (1997) found that overtime, stress-related health problems and depression were becoming increasingly attributable to their post-displacement environment and less on conflict-induced traumatic experiences, citing growing emphasis on arduous living conditions, deep economic trouble, a perceived lack of interest from the Georgian government in their condition, and a growing sense of victimization, stigmatization, isolation, and segregation by local populations frustrated by their continued presence.

JHU (2012) is the only mental health study to dissociate by dwelling type, demonstrating inconsiderable differences in GAD (70.6% v 71.2%) and depression (73.2% v 68.3%) between CC and PA-IDPs, neither of which were significant. Khechuashvili (2014) and SC (2002) are the only studies comparing IDPs to the general population. The latter, conducted in 2000, found that CC-IDPs had greater prevalence of depression symptoms compared to the general population (89.9% v 65.4%). Khechuashvili (2014) was not focused on the prevalence of mental health

disorders but rather the self-perception of psychological well-being. Surprisingly, there were no significant differences observed on the total score and subscales of psychological well-being, or four out of five factors on the posttraumatic growth inventory; IDPs scored lower on the New Possibility factor, which indicates lower confidence in the opening of new possibilities *in general*. Basishvili (2011) and Chikovani (2015) also report the prevalence of insomnia (41.4%), PTSD (23.5%), depression (14.4%), anxiety (10.9%), and comorbidity symptoms (12.7%), but without comparisons between subgroups of IDPs. In addition to age, gender, and status, a number of other demographic characteristics were significantly associated with mental disorder symptoms. Lower levels of education, poorer economic status and community conditions, greater cumulative trauma (experience of more traumatic events), particular traumatic events (witness of murder, physical abuse, among others), being widowed, and having a disability or long-term illness increased the risk of PTSD, depression, anxiety, and somatic distress symptoms by 1.5-10 times (Makhashvili, Comellas, Saxon, Chikovani).

Physical Health

The prevalence of circulatory, respiratory, gastrointestinal, urogenital, skin, and osteo-muscular disease, as well as injuries and disease of blood and blood forming organs were roughly 1.5-4 times higher among IDPs than the GP. Infectious (10.6% of cases), circulatory (19.0%), respiratory (25.7%), digestive (10.1%), and urogenital diseases (8.2%) were also the most frequent health problems facing IDPs in the five year span. Circulatory, respiratory, digestive, urogenital, skin, osteo-muscular, and hematic disease were roughly 2-3 times more prevalent in IDP children. Respiratory (43.8%) and infectious (31.6%) disease were by far the most frequent health problems, although there appears to some regional variation.[27]

Surveys conducted by SC (2000-02) and IFRC (1999) offer a slightly more reliable view of the health status of IDPs in the same time period. IRFC (1999) includes CC- and PA-IDPs as well as a control local population in various regions throughout the country. Similarly, SC (2000-02) compares CC-IDPs in west Georgia (Imereti and Samegrelo) to the local population. Surprisingly, IFCR (1999) found acute and chronic illnesses were reported roughly equally between all three groups (CC-IDP, PA-IDP, and local population. SC (2000-02), on the otherhand, uncovered the opposite trend with CC-IDPs reporting significantly higher proportion of acute (41%) and chronic (33%) illness compared to the GP (33% and 18%, respectively); CC-IDPs also reported higher rates of comorbidity (both acute and chronic illness; 55% vs. 36%). Waterborne infections and diarrhea were the most common cause of health problems were also mentioned by focus groups (SC 2002).[3 27]

ELHRA (2018) found higher rates of chronic illness and poor self-perceived health status relative to non-IDPs. Over 40% of respondents reported being chronically ill, citing cardio-vascular (29%), gastrointestinal (13%), musculoskeletal (13%), and other chronic illnesses (21%). Nearly 16% of respondents rate their health status – defined as the absence of disease or injury as well as overall physical, mental, and social well-being – as poor or very poor.[28]

According to a study conducted by the Medical University of Georgia, high levels of cardiovascular disorders among IDPs may be correlated with the prevalence of PTSD. The study found a strong association between PTSD and hypertension, as well as incidences of stroke and heart attack. [29] Another study in 2001 also showed a clear association between post-traumatic and psychological distress and cardiovascular morbidity among IDPs.[30] Moreover, the high prevalence of infectious diseases is believed to be associated with deteriorated sanitary and hygienic conditions in most of the CCs where IDPs reside.[27]

Using the IHS between 2011-2013, World Bank (2016) found that people with disabilities represented 6.29% of the IDP population in contrast to 4.74% of non-IDPs. The difference, however, is slightly less dramatic over greater time periods. Using the IHS between 2009-2012, 4.75% of the surveyed IDP population reported a disability compared to 4.29% of non-IDPs; between 2013-2018, the gap widens with 4.57% (IDP) and 3.66% (non-IDP). SC (2002) reported a marginally lower percentage of IDP families with one or more family members with a physical disability compared to the GP (14.8% vs. 15.3%).[16] Surprisingly, the UN OCHA (2000) reports a substantial difference in disability, with IDPs over twice as likely to have a physical disability than the GP.[14]

Determinants of HIV/HCV Infection

Substance use – primarily via drug injection – is one of the primary determinants of infection for HIV and HCV[31 32]. Both are blood-borne viruses transmitted through the exchange of contaminated blood. The sharing of needles and the length/frequency of use are factors that affect the risk of infection. Studies document disproportionately higher rates of HIV and HCV among injection drug users both in Georgia and abroad [32 33 34].

The rise in heterosexual transmission, on the otherhand, is likely driven by greater risky sexual activity between drug users and partners. Curatio found condom use at last intercourse varied from 20.8% (Zugdidi) to 45.9% (Telavi), and a relatively small proportion of respondents in all cities reported using condoms with regular partners[34]. As of 2017, the three primary modes of transmission of new cases were injection drug use (23.5%), heterosexual contact (50.3%), and homosexual contact (20.6%), whereas injection continues to be the most common mode of transmission for HCV. [35]

Hazardous substance use also appears to be significantly associated with mental illness. The prevalence of IV drug use appears to be particularly higher in persons with mental illness compared to the general population.[36] Several lines of evidence also support an association between mental illness and HIV/HCV. First, according to a number of seroprevalence studies, the prevalence of HIV, HCV, and other blood-borne infections is higher in persons with mental illness and vice-versa [37 36 38]. Higher rates of HCV infection were recorded in patients with psychosis, histories of significant trauma, depression, PTSD, bipolar disorder, emotional distress, and other psychotic disorders; conditions that are prevalent among CC-IDPs [39]. HCV diagnosis and treatment may also exacerbate mental health issues; patients may suffer from a reactive depression related to excessive fatigue or concerns about their long-term prognosis, and IFN therapy may amplify the symptoms of those with underlying depression [40 41 42]. Nevertheless,

given that studies indicate a high rate of psychiatric comorbidity among alcohol and drug users – with upwards of 80% of patients suffering from a DSM-III lifetime disorder -- it is likely that depressive symptomatology exists before HCV infection in most cases [32].

Several hypotheses attempt to account for these associations. Some symptoms of severe mental illness – particularly cognitive impairment – may contribute directly to an inability to safeguard against the transmission of STDs or blood-borne infections [36]. Studies report that people with severe mental illness frequently engage in high risk behavior for HIV[36]. Depression or substance abuse routinely prevent individuals from accessing treatment or participating in clinical trials, with presumed negative impact on health outcomes [43]. People with severe mental illness also appear to display less knowledge about HIV infection and motivation to engage in protective behaviors, leading them to the belief that they are at little to no risk of contracting HIV [36(p)]. Having multiple partners, unprotected sex, sex with high-risk partners, and in some cases hypersexuality during an acute phase of mental illness, are additional factors that may compromise healthy sexual practices, many of which can similarly be seen among IDUs.[37] Hazardous substance use not only directly correlates to increased risk of infection, but also appears to negatively influence safe sexual practices in some cases. [37]

REFERENCES

1. Ministry of Internally Displaced Persons from the Occupied Territories, Accommodation and Refugees of Georgia. Review of Internally Displaced Persons in Georgia. March 2019.

2. Gogishvili D, Harris-Brandts S. The social and spatial insularity of internally displaced persons: "neighbourhood effects" in Georgia's collective centres. *Cauc Surv*. 2019;7(2):134-156. doi:10.1080/23761199.2019.1617652

3. Dershem L, Gurgenidze N, Holtzman SB. *Poverty and Vulnerability Among Internally Displaced Persons in Georgia: An Update of Their Current Status and Circumstances*. The World Bank; 2002. https://www.researchgate.net/publication/287621578_Poverty_and_Vulnerability_Among_Internally_Displaced_Persons_in_Georgia_An_Update_of_Their_Current_Status_and_Circumstances.

4. *Internally Displaced Persons: A Socio-Economic Survey*. International Federation of the Red Cross (IFRC); 2000.

5. *OCHA Georgia Humanitarian Situation and Strategy 2004*. UN Office for the Coordination of Humanitarian Affairs https://reliefweb.int/report/georgia/ocha-georgia-humanitarian-situation-and-strategy-2004.

6. *Protection of Internally Displaced Persons in Georgia: A Gap Analysis*. United Nations High Commissioner for Refugees; 2009. https://www.unhcr.org/4ad827f59.pdf.

7. Tarkhan-Mouravi G. *Assessment of IDP Livelihoods in Georgia: Facts and Policies*. United Nations High Commissioner for Refugees; 2009. https://www.unhcr.org/4ad827b12.pdf.

8. *The Situation of Human Rights and Freedoms in Georgia*. The Public Defender (Obudsman) of Georgia; 2004. http://www.ombudsman.ge/eng/saparlamento-angarishebi.

9. *Report of the Ministry of Internally Displaced Persons from the Occupied Territories, Accommodation, and Refugees of Georgia*. Ministry of Internally Displaced Persons from the Occupied Territories, Accommodation, and Refugees of Georgia; 2013. http://mra.gov.ge/geo/static/825.

10. *Food Security, Child Nutrition, and Agricultural Livelihoods of Conflict-Affected Persons in Georgia*. Food and Agriculture Organization, United Nations Children's Fund, World Food Program; 2009. https://documents.wfp.org/stellent/groups/public/documents/ena/wfp211532.pdf.

11. Hovey G, de Berry J, Huybens E. *Supporting the Livelihoods of Internally Displaced Persons in Georgia: A Review of Current Practices and Lessons Learned*. The World Bank; 2013.

http://documents.worldbank.org/curated/en/859881468236363043/pdf/791740WP0Georg0Box0377356B00PUBLIC0.pdf.

12. Kurshitashvili N. The Impact of Socially Ir/responsible Resettlement on the Livelihoods of Internally Displaced Persons in Georgia. *Refug Surv Q*. 2012;31(2):98-118. doi:10.1093/rsq/hds001

13. *Accountability in Aid Delivery - The Renovation of Collective Centers for Georgia's Internally Displaced Persons*. Transparency International Georgia; 2011. https://docs.google.com/viewerng/viewer?url=https://www.transparency.ge/sites/default/files/accountabilityinaiddelivery_eng.pdf.

14. *Profile of Internal Displacement: Georgia Compilation of the Information Available in the Global IDP Database of the Norwegian Refugee Council*. Norwegian Refugee Council; 2004. https://www.refworld.org/pdfid/406424744.pdf.

15. *Intentions Survey on Durable Solutions: Voices of Internally Displaced Persons in Georgia*. United Nations High Commissioner for Refugees; 2015. https://www.refworld.org/pdfid/55e575924.pdf.

16. Dershem L, Tskitishvili G, Kechakmadze V. *Social Capital and Employment Opportunities Among Internally Displaced Persons (IDPs) in Georgia*. The World Bank; 2005. https://www.researchgate.net/publication/287632644_Social_Capital_and_Employment_Opportunities_Among_Internally_Displaced_Persons_IDPs_in_Georgia.

17. Badurashvili I, Nadareishvili M. Social Impact of Emigration and Rural-Urban Migration in Central and Eastern Europe Final Country Report Georgia April 2012. In: ; 2012.

18. Frichova Grono M. *Displacement in Georgia: IDP Attitudes to Conflict, Return and Justice*. Conciliation Resources; 2011. https://reliefweb.int/sites/reliefweb.int/files/resources/Full_Report_556.pdf.

19. *Baseline Survey of the IDP Settlements and Their Neighbouring Communities in Kvemo Kartli and Shida Kartli*. CARE International Caucasus; 2009. http://www.geowel.org/files/idp_research_geowel_2010_full_eng.pdf.

20. Salukvadze J, Gachechiladze R, Gogishvili D, et al. *Coping with Marginality and Exclusion: Can Refugees Communities Successfully Integrate into Mainstream Urban Societies in Georgia?* Tbilisi State University; 2013. https://www.researchgate.net/profile/David_Gogishvili/project/Coping-with-Marginality-and-Exclusion-Can-IDP-Internally-Displaced-Person-Communities-Successfully-Integrate-into-Mainstream-Urban-Societies-in-Georgia/attachment/5864143608ae73e55b21544e/AS:444354524389376@1482953782230/download/Scientific_Report_IDP_Project.pdf?context=ProjectUpdatesLog.

21. Gogishvili D. *Urban Dimensions of Internal Displacement in Georgia: The Phenomenon and the Emerging Housing Policy*. Rochester, NY: Social Science Research Network; 2015. https://papers.ssrn.com/abstract=2591291. Accessed July 18, 2019.

22. Tukhashvili M, Tsartsidze M, Antadze T, Latsabidze N, Shelia M, Toria M. *Internally Displaced Persons in the Georgian Labour Market*. Tbilisi: Universal; 2012. https://www.researchgate.net/publication/293783116_INTERNALLY_DISPLACED_PERSONS_IN_THE_GEORGIAN_LABOUR_MARKET.

23. Chelidze N. *Asylum Seekers, Refugees and Internally Displaced Persons (IDPs) in Georgia: The Challenges of Social Cohesion*. Consortium for Applied Research on International Migration http://www.carim-east.eu/media/exno/Explanatory%20Notes_2013-130.pdf.

24. Tomlinson F, Egan S. From Marginalization to (Dis)Empowerment: Organizing Training and Employment Services for Refugees. *Hum Relat*. 2002;55(8):1019-1043. doi:10.1177/0018726702055008182

25. *Georgia: IDPs in Georgia Still Need Attention a Profile of the Internal Displacement Situation*. Norwegian Refugee Council; 2009.

26. Lacroix R, Benner H, Ashwill M, et al. *Gender Based Violence in Georgia: Links Among Conflict, Economic Opportunities and Services*. The World Bank; 2017. http://www.parliament.ge/uploads/other/77/77112.pdf.

27. Zoidze A, Djibuti M. *IDP Health Profile Review in Georgia*. United Nations Development Programme in Georgia; 2004. https://www.researchgate.net/publication/256838902_IDP_Health_Profile_in_Georgia.

28. *Health Service Utilization, Expenditures and Health Status among IDP Population in Georgia*. Enhancing Learning and Research for Humanitarian Assistance https://www.elrha.org/wp-content/uploads/2018/01/HRU-Survey-Report.pdf.

29. Gvalia M, Khorava D, Odisharia M. The Impact of Risk Factors on the Incidence of Arterial Hypertension Among the IDPs. *Georgian Med News*. 2003;2(95).

30. Khorava D, Daniela M. Main Demographic Trends and Health Status of IDPs from Abkhazia. *J Health Sci Manag Public Health*. 2002;3.

31. Rourke SB, Sobota M, Tucker R, et al. Social determinants of health associated with hepatitis C co-infection among people living with HIV: results from the Positive Spaces, Healthy Places study. *Open Med*. 2011;5(3):e120-e131.

32. Johnson ME, Fisher DG, Fenaughty A, Theno SA. Hepatitis C virus and depression in drug users. *Am J Gastroenterol*. 1998;93(5):785-789. doi:10.1016/S0002-9270(98)00105-1

33. Hagan LM, Kasradze A, Salyer SJ, et al. Hepatitis C prevalence and risk factors in Georgia, 2015: setting a baseline for elimination. *BMC Public Health*. 2019;19(3):480. doi:10.1186/s12889-019-6784-3

34. Shengelia N, Chikovani I, Sulaberidze L, Sirbiladze T, Tavzarashvili L. *HIV Risk and Prevention Behaviors among People Who Inject Drugs in Seven Cities of Georgia, Bio-Behavioral Surveillance Survey in 2017*.; 2017. doi:10.13140/RG.2.1.3570.8400

35. *Health Care Statistical Yearbook 2017 Georgia*. National Center for Disease Control and Public Health; 2017. http://www.ncdc.ge/Handlers/GetFile.ashx?ID=114b7ef6-0fa1-424a-9c01-6af08ffa63cc.

36. Essock SM, Dowden S, Constantine NT, et al. Blood-Borne Infections and Persons With Mental Illness: Risk Factors for HIV, Hepatitis B, and Hepatitis C Among Persons With Severe Mental Illness. *Psychiatr Serv*. 2003;54(6):836-841. doi:10.1176/appi.ps.54.6.836

37. Hughes E, Bassi S, Gilbody S, Bland M, Martin F. Prevalence of HIV, hepatitis B, and hepatitis C in people with severe mental illness: a systematic review and meta-analysis. *Lancet Psychiatry*. 2016;3(1):40-48. doi:10.1016/S2215-0366(15)00357-0

38. Batista-Neves SC, Quarantini LC, de Almeida AG, et al. High frequency of unrecognized mental disorders in HCV-infected patients. *Gen Hosp Psychiatry*. 2008;30(1):80-82. doi:10.1016/j.genhosppsych.2007.08.014

39. el-Serag HB, Kunik M, Richardson P, Rabeneck L. Psychiatric disorders among veterans with hepatitis C infection. *Gastroenterology*. 2002;123(2):476-482. doi:10.1053/gast.2002.34750

40. Malhotra S, Kaur N, Kumar P, Bhatia M, Hans C. Hepatitis C and Depression. *Delhi Psychiatry J*. 2011;14(1).

41. Fortier E, Alavi M, Bruneau J, et al. Depression, Anxiety, and Stress Among People With Chronic Hepatitis C Virus Infection and a History of Injecting Drug Use in New South Wales, Australia. *J Addict Med*. 2017;11(1):10-18. doi:10.1097/ADM.0000000000000261

42. Nelligan J, Indest DW, Hauser P, et al. Mental Health & Hepatitis C. In: *Hepatitis C Choices Diverse Viewpoints and CHoices for Your Hepatitis C Journey*. 4th ed. Caring Ambassadors Program; 2008. https://hepcchallenge.org/wp-content/uploads/2017/09/Hepatitis_C_Choices_4thEdition.pdf.

43. Sublette VA, Douglas MW, McCaffery K, George J, Perry KN. Psychological, lifestyle and social predictors of hepatitis C treatment response: a systematic review. *Liver Int*. 2013;33(6):894-903. doi:10.1111/liv.12138
